# Association of Brain Metastases With Survival in Patients With Limited or Stable Extracranial Disease: A Systematic Review and Meta-analysis

**DOI:** 10.1001/jamanetworkopen.2023.0475

**Published:** 2023-02-23

**Authors:** Alyssa Y. Li, Karolina Gaebe, Amna Zulfiqar, Grace Lee, Katarzyna J. Jerzak, Arjun Sahgal, Steven Habbous, Anders W. Erickson, Sunit Das

**Affiliations:** 1 Institute of Medical Science, Faculty of Medicine, University of Toronto, Toronto, Ontario, Canada; 2 Division of Medical Oncology, Sunnybrook Health Sciences Centre, Toronto, Ontario, Canada; 3 Department of Radiation Oncology, Sunnybrook Health Sciences Centre, Toronto, Ontario, Canada; 4 Ontario Health (Cancer Care Ontario), Toronto, Ontario, Canada; 5 Epidemiology and Biostatistics, Western University, London, Ontario, Canada; 6 Department of Laboratory Medicine and Pathobiology, Faculty of Medicine, University of Toronto, Toronto, Ontario, Canada; 7 Division of Neurosurgery, Department of Surgery, University of Toronto, St Michael’s Hospital, Toronto, Ontario, Canada

## Abstract

**Importance:**

Intracranial metastatic disease (IMD) is a severe complication of cancer with profound prognostic implications. Patients with IMD in the setting of limited or stable extracranial disease (IMD-SE) may represent a unique and understudied subset of patients with IMD with superior prognosis.

**Objective:**

To evaluate overall survival (OS), progression-free survival (PFS), and intracranial PFS (iPFS) in patients with IMD-SE secondary to any primary cancer.

**Data Sources:**

Records were identified from MEDLINE, EMBASE, CENTRAL, and gray literature sources from inception to June 21, 2021.

**Study Selection:**

Studies in English reporting OS, PFS, or iPFS in patients with IMD-SE (defined as IMD and ≤2 extracranial metastatic sites) and no prior second-line chemotherapy or brain-directed therapy were selected.

**Data Extraction and Synthesis:**

Author, year of publication, type of study, type of primary cancer, and outcome measures were extracted. Random-effects meta-analyses were performed to estimate effect sizes, and subgroup meta-analysis and metaregression were conducted to measure between-study differences in February 2022.

**Main Outcomes and Measures:**

The primary end point was OS described as hazard ratios (HRs) and medians for comparative and single-group studies, respectively. Secondary end points were PFS and iPFS.

**Results:**

Overall, 68 studies (5325 patients) were included. IMD-SE was associated with longer OS (HR, 0.52; 95% CI, 0.39-0.70) and iPFS (HR, 0.63; 95% CI, 0.52-0.76) compared with IMD in the setting of progressive extracranial disease. The weighted median OS estimate for patients with IMD-SE was 17.9 months (95% CI, 16.4-22.0 months), and for patients with IMD-PE it was 8.0 months (95% CI, 7.2-12.8 months). Pooled median OS for all patients with IMD-SE was 20.9 months (95% CI, 16.35-25.98 months); for the subgroup with breast cancer it was 20.2 months (95% CI, 10.43-38.20 months), and for non–small cell lung cancer it was 27.5 months (95% CI, 18.27-49.66 months). Between-study heterogeneity for OS and iPFS were moderate (*I^2^* = 56.5%) and low (*I^2^* = 0%), respectively.

**Conclusions and Relevance:**

In this systematic review and meta-analysis of patients with IMD-SE, limited systemic disease was associated with improved OS and iPFS. Future prospective trials should aim to collect granular information on the extent of extracranial disease to identify drivers of mortality and optimal treatment strategies in patients with brain metastases.

## Key Points

**Question:**

What are the clinical outcomes of patients with intracranial metastatic disease in the setting of stable extracranial disease (IMD-SE) compared with patients with brain metastases and progressive extracranial disease (IMD-PE)?

**Findings:**

This meta-analysis of 68 studies found prolonged overall survival in patients with IMD-SE compared with those with IMD-PE. Pooled median overall survival for patients with IMD-SE was 20.9 months, and weighted median overall survival was 17.9 months for patients with IMD-SE and 8.0 months for patients with IMD-PE.

**Meaning:**

These results suggest that IMD-SE represents a subgroup of patients with intracranial metastatic disease who have favorable survival.

## Teaser

This meta-analysis evaluates overall survival, progression-free survival, and intracranial progression-free survival in patients with stable vs progressive intracranial metastatic disease secondary to any primary cancer.

## Introduction

Intracranial metastatic disease (IMD) is a serious complication of cancer, arising in 5% to 20% of patients with breast cancer, 20% to 56% of patients with lung cancer, and 7% to 16% of patients with melanoma.^1^ Patients with IMD experience reduced overall survival (OS) compared with those without brain metastases, with a historic median survival of 3.6 to 3.8 months following diagnosis.^2^ The development of IMD is therefore one of the primary survival-limiting factors in patients with cancer.

The prognostic impact of IMD is influenced by patient- and disease-specific factors, including intracranial disease burden, as survival in patients with at least 2 or 3 brain metastases has been found to be reduced compared with those with fewer brain metastases.^3^ The utility of intracranial disease as an independent predictor of OS is also reflected in several prognostic tools, including disease-specific graded prognostic assessment (ds-GPA).^4^ However, the notion that intracranial disease burden drives mortality in patients with IMD has been challenged by recent studies that demonstrate similar OS in patients with 4 to 10 brain metastases and less than 3 or 4 brain metastases.^5^

With advances in systemic therapies, an increasing number of patients develop brain metastases despite stable or absent systemic disease.^6^ There is evidence to suggest that the absence of extracranial disease (ECD) is associated with prolonged OS and is, therefore, also incorporated into the ds-GPA.^4^ However, metastatic illness exists on a spectrum from limited metastases to disseminated disease, and this continuum is not captured in prognostic tools based on the binary presence or absence of ECD.^7^ There is a need to further characterize the impact of limited or stable ECD on the survival of patients with IMD.

In this study, we hypothesized that there may exist a subpopulation of patients with IMD in the setting of stable or limited ECD (IMD-SE) who experience prolonged OS, compared with patients with IMD and progressive or unstable ECD (IMD-PE). We performed a systematic review and meta-analysis to evaluate OS, progression-free survival (PFS), and intracranial PFS (iPFS) in patients with IMD-SE compared with patients with IMD-PE.

## Methods

This systematic review and meta-analysis was conducted in accordance with the Preferred Reporting Items for Systematic Reviews and Meta-analyses (PRISMA) reporting guideline and the Meta-analysis of Observational Studies in Epidemiology (MOOSE) reporting guideline and was registered in PROSPERO (CRD42021261563). Study protocols and amendments can be accessed on the Open Science Framework (OSF).

### Search Strategy and Study Eligibility

A literature search was performed in MEDLINE, EMBASE, CENTRAL, and gray literature sources on June 21, 2021. MeSH terms included *stable*, control*, and *brain metastases*. Relevant gray literature sources and search query are available on OSF (and in eMethods and eTable 1 in Supplement 1). All years from database inception to the search date were included. Only articles and abstracts published in English were considered due to resource constraints. Reference lists of all included studies were scanned to ensure saturation.

Eligible studies reported OS in patients aged at least 18 years with IMD-SE, which we defined as the presence of brain metastases, 0 to 2 extracranial metastatic sites, and no prior second-line chemotherapy or second-line brain-directed therapy, which may reflect active systemic disease, without constraints on the type of first-line brain-directed therapy received, cancer type, IMD burden, or ECD location. Studies that reported on patients with IMD and controlled ECD without further detailing ECD extent or prior treatment were included. Author definitions for ECD control and stability are described in eTable 2 in Supplement 1). In our primary analysis, these patients were classified as having IMD-SE. In sensitivity analyses, these studies were excluded. In some studies, 2 separate cohorts were reported: a cohort of patients with IMD-SE according to our criteria, and a second cohort of patients described as having controlled ECD without detailing extracranial metastatic burden or prior treatment (ie, not explicitly meeting our criteria of IMD-SE). In these circumstances, data from the IMD-SE cohort was used for the primary analysis, whereas data from the controlled ECD cohort was used for secondary analyses comparing patients with controlled ECD with those with uncontrolled ECD as defined by study authors.

IMD-PE was defined as IMD with unstable, disseminated ECD, or otherwise not fulfilling the aforementioned criteria of IMD-SE. Case reports, case series, and review articles were excluded. Title-and-abstract and full-text screening were performed in duplicate by 3 reviewers (A.Y.L., K.G., A.Z.).

### Data Extraction and Quality Assessment

Data extraction was completed in duplicate by 4 reviewers (A.Y.L., K.G., A.Z., G.L.). Conflicts were resolved through discussion. Corresponding authors were not contacted given resource constraints. Extracted data are available in the Supplement (eMethods in Supplement 1). Quality assessment of observational studies was performed using the Newcastle-Ottawa Scale. Quality assessment of randomized clinical trials (RCTs) was performed using the Cochrane Risk of Bias 2 (RoB 2) tool.

### Statistical Analysis

The primary end point was OS, defined as the length of time from IMD diagnosis until death or loss to follow-up unless otherwise specified and extracted as medians and unadjusted hazard ratios (HR) for single-group and comparative studies, respectively. Secondary end points included iPFS.

Random-effects meta-analyses using the Paule-Mandel estimator pooled HRs with 95% CI for OS and iPFS between IMD-SE and IMD-PE cohorts.^8^ In studies which displayed Kaplan-Meier curves but reported no effect size estimates for OS and iPFS in formats amenable to pooling, median OS and iPFS were derived according to Guyot et al^9^ and used calculate HRs.^9^ Subgroup analyses were performed for primary cancer type, Agency for Healthcare Research and Quality (AHRQ) rating, IMD-SE definition, and whether HRs were reported or derived from published Kaplan-Meier curves.

Metaregression was performed to assess the association of cohort size, publication year, and risk of bias assessment with the summary effect size. A secondary analysis pooled distribution-free single-group survival outcomes in IMD-SE, stratified by primary cancer type and OS index date, using the method by Combescure et al^10^ to generate summary survival curves. Weighted median OS, iPFS, and PFS were estimated using the method reported by McGrath et al.^11^ All statistical analyses were performed from January to February 2022 using R version 4.0.3 (R Project for Statistical Computing) and meta,^12^ metafor,^13^ metaSurvival,^14^ and metamedian^11^ packages (eMethods in Supplement 1). Two-sided *P* < .05 was considered statistically significant.

Statistical heterogeneity was assessed using outlier identification, Baujat plots, leave-one-out analysis, and graphic display of study heterogeneity (GOSH) statistics^15,16,17^ in addition to *I*^2^ and *Q* statistics.^18^ Egger tests and funnel plot inspection were performed to assess for publication bias.^19^

## Results

### Study Characteristics

**Figure 1.**
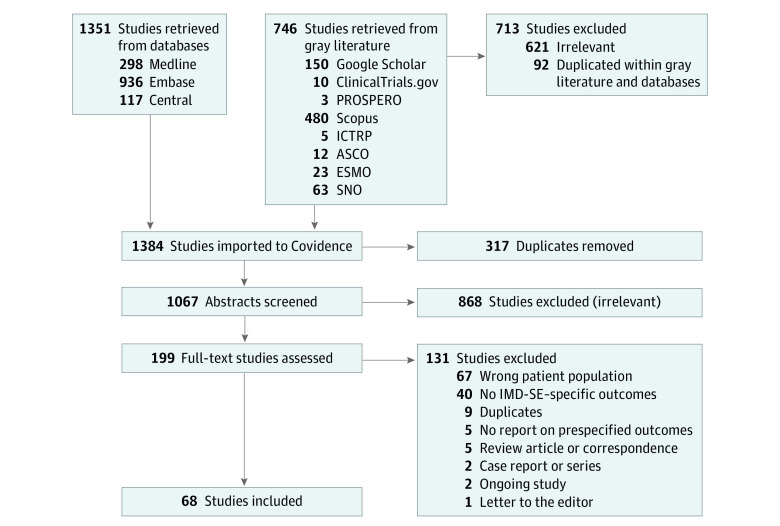
Study Selection ASCO indicates American Society of Clinical Oncology; ESMO, European Society for Medical Oncology; ICTRP, International Clinical Trials Registry Platform; IMD-SE, intracranial metastatic disease in the setting of stable extracranial disease; SNO, Society for Neuro-Oncology.

The literature search identified 1067 unique studies, of which 68 studies met eligibility criteria (Figure 1; eTable 3 in Supplement 1).^3,20,21,22,23,24,25,26,27,28,29,30,31,32,33,34,35,36,37,38,39,40,41,42,43,44,45,46,47,48,49,50,51,52,53,54,55,56,57,58,59,60,61,62,63,64,65,66,67,68,69,70,71,72,73,74,75,76,77,78,79,80,81,82,83,84,85,86^ Among included studies, there were 58 retrospective cohort studies, 7 prospective cohort studies, and 3 RCTs. Thirty-four reported on non–small cell lung cancer (NSCLC), 3 on breast cancer, 2 on small-cell lung cancer, 1 on melanoma, and 1 reported multiple lung cancers. Twenty-five studies reported multiple primary cancers, and 2 did not specify primary cancer type. There were 5325 patients with IMD-SE, 4822 meeting our IMD-SE criteria and 503 with stable ECD as defined by study authors, and 1466 patients with IMD-PE were included across all studies; the number of patients with IMD-SE and IMD-PE were not reported in 2 studies.^28,31^ Median follow-up was reported in 42 studies and ranged from 4.7 to 102 months (eTable 3 in Supplement 1).

### OS in Patients With IMD-SE vs Patients With IMD-PE

**Figure 2.**
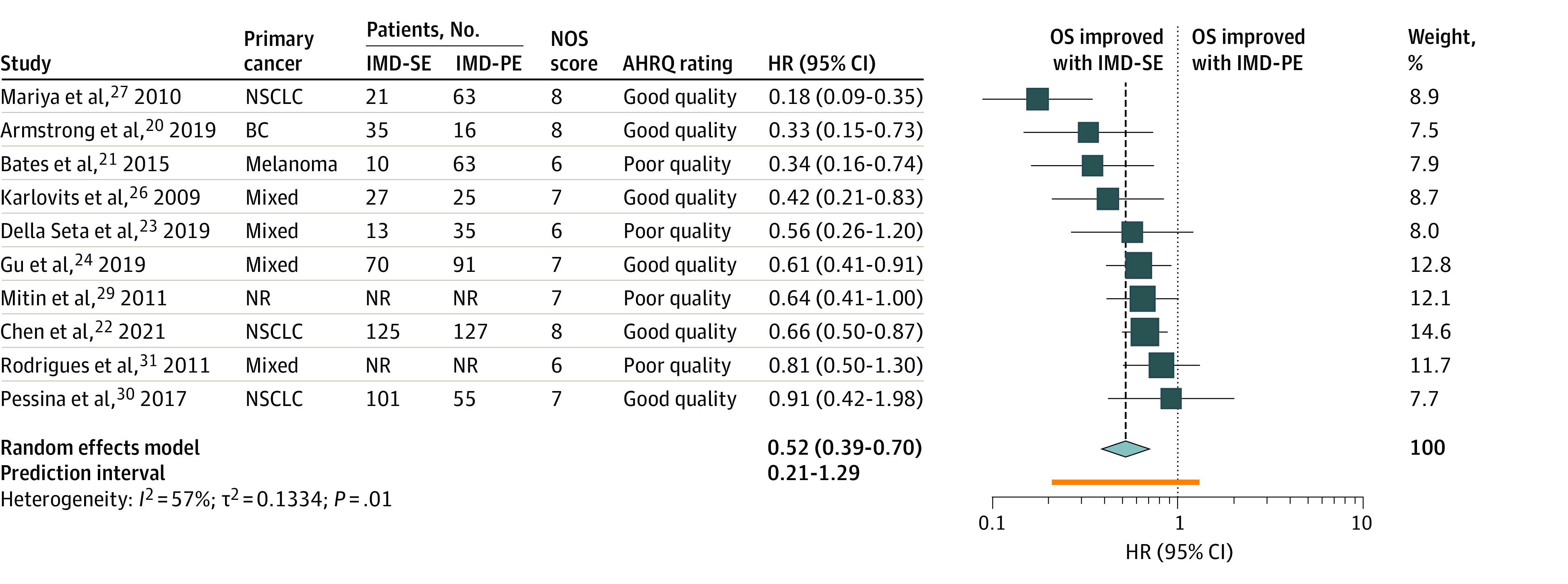
Random-Effects Meta-analysis of the Primary Outcome of OS in Patients With IMD-SE Vs Patients With IMD-PE The size of the squares is proportional to the weight of the study. The light blue diamond represents the pooled estimate within a 95% CI, while the horizontal lines indicate the 95% CI of each study. The orange bar represents the prediction interval, and the vertical dotted line is the pooled estimated HR. The number of patients with IMD-SE and IMD-PE were not reported in 2 studies and were not included in the total number of patients.^28,31^ AHRQ indicates Agency for Healthcare Research and Quality; BC, breast cancer; HR, hazard ratio; IMD-PE, intracranial metastatic disease in the context of progressive extracranial disease; IMD-SE, intracranial metastatic disease in the context of stable extracranial disease; NOS, Newcastle-Ottawa Scale; NR, not reported; NSCLC, non–small cell lung cancer; OS, overall survival.

Twelve studies compared HR for OS between IMD-SE and IMD-PE,^20,21,22,23,24,25,26,27,28,29,30,31^ of which 10 were eligible for meta-analysis.^20,21,22,23,24,26,27,28,30,31^ Four reported patients with controlled ECD without details on extracranial metastatic burden or prior treatment.^20,27,28,31^ IMD-SE was associated with prolonged OS compared with IMD-PE (HR, 0.52; 95% CI, 0.39-0.70; n = 877 patients) (Figure 2; eTable 4 in Supplement 1). Subgroup analysis found no significant differences in OS between the 4 studies reporting on patients with controlled ECD (HR, 0.45; 95% CI, 0.07-0.73; n = 135 patients)^20,27,28,31^ and the 6 studies that satisfied our criteria for IMD-SE (HR, 0.57; 95% CI, 0.38-0.84; n = 742 patients) (*P* = .47).^21,22,23,24,26,30^ The weighted median OS estimate for patients with IMD-SE was 17.9 months (95% CI, 16.4-22.0 months; n = 49 studies; n = 3229 patients), and for patients with IMD-PE it was 8.0 months (95% CI, 7.2-12.8 months; n = 9 studies; n = 625 patients) (Table).

**Table.** Weighted Median OS, iPFS, and PFS Estimates in Months

Population	No. of studies	No. of patients	Estimate, mo	
			Outcome measure	Median (95% CI)
IMD-SE	49^a^	3229	OS	17.9 (16.4-22.0)
	4^b^	365	iPFS	13.6 (5.0-13.6)
	12^c^	935	PFS	7.0 (4.6-12.0)
IMD-PE	9^d^	625	OS	8.0 (7.2-12.8)
	3^e^	519	iPFS	5.5 (1.9-5.5)
Controlled ECD	6^f^	360	OS	15.2 (10.1-28.1)
Uncontrolled ECD	4^g^	268	OS	8.0 (6.0-8.0)

Abbreviations: ECD, extracranial disease; IMD-PE, intracranial metastatic disease in the setting of progressive ECD; IMD-SE, intracranial metastatic disease in the setting of stable ECD; iPFS, intracranial progression-free survival; OS, overall survival; PFS, progression-free survival.

^a^ Data are from 49 studies.^3,20,21,24,26,27,29,30,32,33,34,35,36,38,39,40,41,42,43,44,47,49,50,51,52,53,55,58,59,60,61,63,64,66,67,68,69,71,72,73,78,80,81,83,84,85,86^

^b^ Data are from 4 studies.^3,21,65,84^

^c^ Data are from 12 studies.^50,52,58,59,60,63,66,69,72,78,80,86^

^d^ Data are from 9 studies.^3,20,21,24,26,27,29,30,47^

^e^ Data are from 3 studies.^3,21,65^

^f^ Data are from 6 studies.^3,20,27,30,47,61^

^g^ Data are from 4 studies.^3,20,27,47^

### OS in IMD in the Setting of Controlled ECD vs Uncontrolled ECD

Of 68 studies, 8 reported on a single-group cohort of patients with IMD and stable or controlled ECD without further detailing extracranial metastatic burden or prior treatment.^3,20,27,28,31,47,56,61^ Two studies each reported separate patient cohorts that met our criteria of IMD-SE and another with stable or controlled ECD.^24,30^ Five studies compared OS in the setting of controlled vs uncontrolled ECD and were amenable to meta-analysis (HR, 0.47; 95% CI, 0.28-0.80; n = 135 patients; eTable 5 and eFigure 1 in Supplement 1).^20,24,27,28,31^ Weighted median OS estimates were 15.2 months (95% CI, 10.1-28.1 months; n = 360 patients; n = 6 studies) for patients with IMD in the setting of controlled ECD without further details on extracranial metastatic burden or prior treatment and 8.0 months (95% CI, 6.0-8.0 months; n = 268 patients; n = 4 studies) in patients with IMD in the setting of uncontrolled ECD (Table).

### Single-Group OS in Patients With IMD-SE

**Figure 3.**
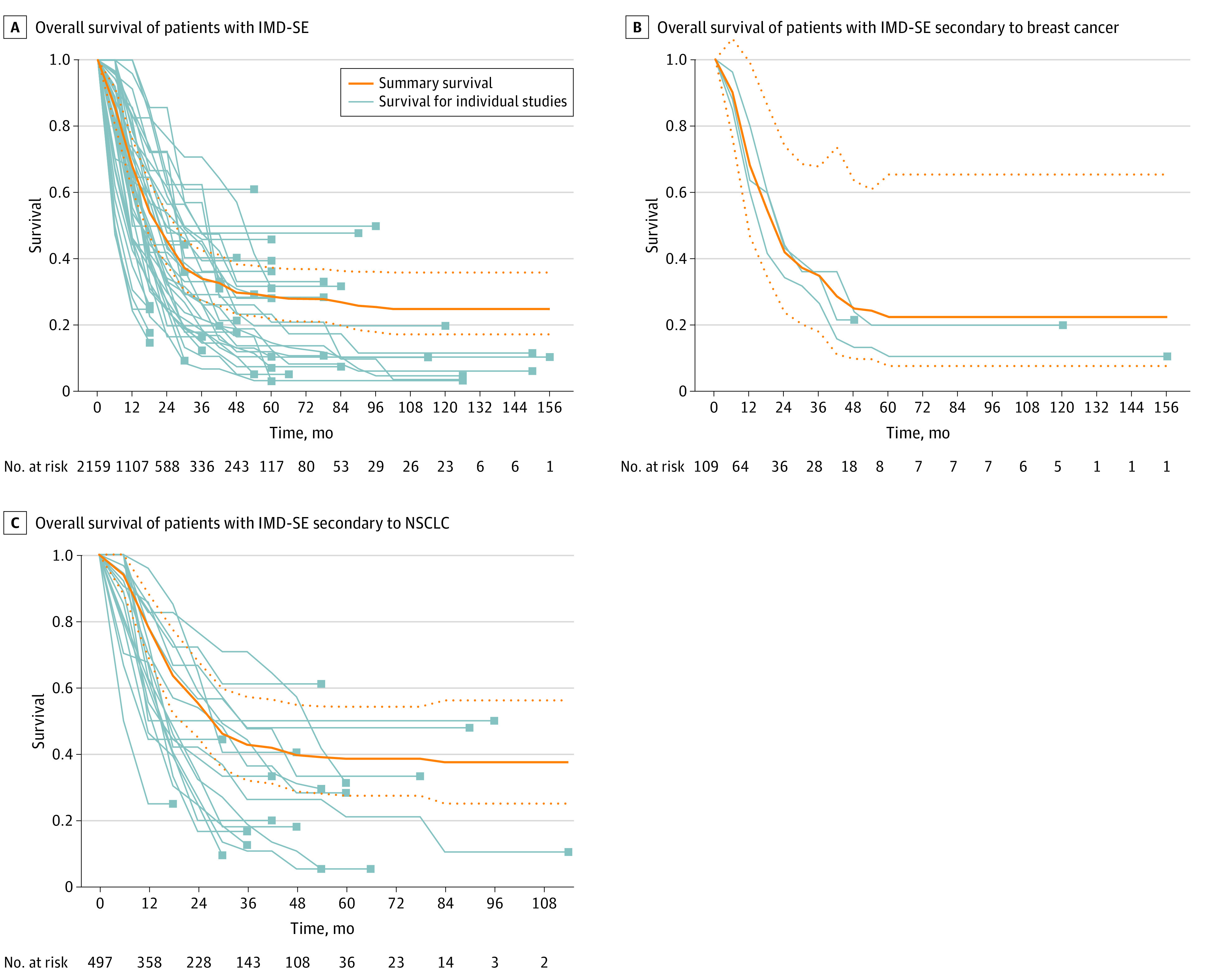
Pooled Summary Overall Survival (OS) of Patients With IMD-SE Light gray lines represent OS curves for individual studies. The solid orange lines represent the summary survival curves, and the dashed orange lines represent 95% CI. IMD-SE, intracranial metastatic disease in the context of stable extracranial disease; NSCLC, non–small cell lung cancer.

Twenty-seven studies reported median OS in patients with IMD-SE.^20,26,27,32,33,34,36,39,41,42,43,44,49,55,59,60,61,63,66,67,71,72,73,80,81,85,86^ Among these, the pooled median OS was 20.9 months; (95% CI, 16.35-25.98 months; n = 2159 patients) for all patients with IMD-SE (Figure 3A). Of note, 9 studies did not report a median follow-up.^20,34,39,55,59,60,63,73,80^ In patients with IMD-SE secondary to breast cancer^20,60^ and NSCLC,^27,36,39,41,42,43,44,59,63,66,73,81,85^ pooled median OS were 20.2 months (95% CI, 10.4-38.2 months; n = 2 studies; n = 109 patients) (Figure 3B) and 27.5 months (95% CI, 18.3-49.7 months; n = 13 studies; n = 497 patients) (Figure 3C), respectively. An estimated 23 patients across 4 studies (1.1% of 2159 patients with IMD-SE) survived beyond 10 years (Figure 3A).^32,49,60,67^

Weighted median OS from any first-line treatment was 23.5 months (95% CI, 12.7-37.5 months; n = 27 studies; n = 675 patients) (eFigure 2A in Supplement 1), whereas the weighted median OS from first-line treatment of brain metastases was 16.9 months (95% CI, 8.8-29.4 months; n = 6 studies; n = 566 patients) (eFigure 2B in Supplement 1). Weighted median OS from brain metastasis diagnosis was 17.0 months (95% CI, 11.6-25.7 months; n = 5 studies; n = 269 patients) (eFigure 2C in Supplement 1).

### Study Quality and Between-Study Heterogeneity on OS for IMD-SE vs IMD-PE

Improved OS was associated with IMD-SE in patients with breast cancer, NSCLC, and melanoma compared with IMD-PE secondary to these cancer types (breast cancer: HR, 0.33; 95% CI 0.10-1.13; NSCLC: HR, 0.48; 95% CI, 0.25-0.91; melanoma: HR, 0.34; 95% CI, 0.10-1.16), with no significant differences between cancer type (*P* = .84). Subgroup analysis similarly did not show significant differences among studies with high vs low risk of bias as determined by AHRQ ratings. Metaregression failed to identify associations between the effect size and number of patients, publication year, or risk of bias.

Of the 3 RCTs included, 1 was at low risk of bias,^34^ while 2 demonstrated some concerns (eFigures 3 and 4 in Supplement 1).^33,86^ The risk of bias assessment for observational studies is reported in eFigures 5, 6, 7, and 8 in Supplement 1. Eight comparative studies were deemed good quality,^20,22,24,25,26,27,29,30^ and the remaining 4 comparative studies were deemed poor quality per AHRQ.^21,23,28,31^

Between-study heterogeneity was moderate (*I^2^* = 56.5%), and influence analysis based on Baujat plot, leave-one-out meta-analysis, and GOSH statistics only identified 1 study, Mariya et al,^27^ as individually contributing to between-study heterogeneity (eFigures 9, 10, and 11 in Supplement 1). In a sensitivity analysis omitting this study, heterogeneity was reduced overall (*I^2^* = 4.4%), while direction and statistical significance of the summary effect size remained unchanged (HR, 0.61; 95% CI, 0.51-0.73; n = 793 patients; (eFigure 12 in Supplement 1). Visual inspection of the funnel plot and Egger test did not suggest publication bias (eFigure 13 in Supplement 1).

### Intracranial Progression-Free Survival Among Patients With IMD-SE or IMD-PE

**Figure 4.**
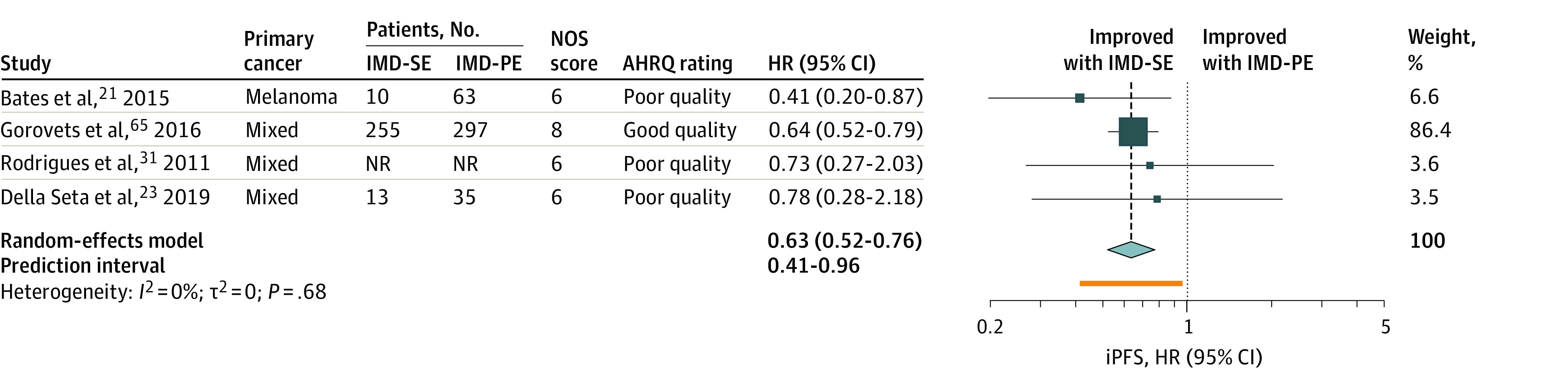
Random-Effects Meta-analysis of iPFS in Patients With IMD-SE Compared With Patients With IMD-PE The size of the squares is proportional to the weight of the study. The light blue diamond represents the pooled estimate within a 95% CI, while the horizontal lines indicate the 95% CI of each study. The orange bar represents the prediction interval, and the vertical dotted line is the pooled estimated HR. The number of patients with IMD-SE and IMD-PE were not reported in one study and were not included in the total number of patients.^31^ AHRQ indicates Agency for Healthcare Research and Quality; HR, hazard ratio; IMD-PE, intracranial metastatic disease in the context of progressive extracranial disease; IMD-SE, intracranial metastatic disease in the context of stable extracranial disease; iPFS, intracranial progression-free survival; NOS, Newcastle-Ottawa Scale; NR, not reported.

Prolonged iPFS was associated with presence of IMD-SE (HR, 0.63; 95% CI, 0.52-0.76; n = 4 studies; n = 673) (Figure 4; eTable 6 in Supplement 1). The weighted median iPFS estimate was 13.6 months (95% CI, 5.0-13.6 months; n = 4 studies; n = 365 patients)^21,23,31,65^ for patients with IMD-SE and 5.5 months (95% CI, 1.9-5.5; n = 3 studies; n = 519 patients)^3,21,65^ patients with IMD-PE (Table). Between-study heterogeneity was low (*I^2^* = 0%) and no outliers were identified.

### Progression-Free Survival in IMD-SE

The weighted median PFS of patients with IMD-SE secondary to any primary cancer was 7.0 months; (95% CI, 4.6-12.0 months; n = 12 studies; n = 935 patients). The Table provides additional details.

## Discussion

This systematic review and meta-analysis captures a clear divergence of OS in patients with IMD and stable ECD from those with uncontrolled ECD, further supporting the notion that OS in patients with IMD may be limited by ECD progression.^28^ To our knowledge, this is the first meta-analysis to delineate the clinical outcomes of patients with IMD-SE and emphasize the importance of ECD status on survival in patients with IMD. For example, Mariya et al^27^ reported a median OS of 32 months in NSCLC patients with IMD-SE compared with just 7 months in those with IMD-PE (HR, 0.28; 95% CI, 0.12-0.64; *P* = .003). These observations are reminiscent of the concept of oligometastasic disease, an intermediate state in which metastatic progression is seen in the setting of primary disease control.^7,87^ Although oligometastatic IMD has historically been defined as a state of limited intracranial disease burden (less than or equal to 4 brain metastases^76^ or 1 to 5 lesions amenable to local therapy, per ESTRO-ASTRO^87^), our results suggest a need to reevaluate this definition to incorporate ECD stability.

In this meta-analysis, 5 studies incorporated intracranial disease burden in their study design. Della Seta et al^23^ found improved OS with absent ECD compared with present ECD in patients with a single brain metastasis secondary to NSCLC or melanoma (HR, 0.48; 95% CI, 0.28-0.81; *P* = .005). Prolonged OS was also reported in patients with less than or equal to 4 brain metastases secondary to NSCLC without ECD compared with those with ECD by Pessina et al^26^ (HR, 0.91; 95% CI, 0.48-2.27) and Karlovits et al^30^ (HR, 0.42; 95% CI, 0.21-0.83; *P* = .01). Rodrigues et al^31^, which reported prolonged OS in patients without ECD compared with those with ECD, included patients with up to 7 brain metastases (HR, 0.81; 95% CI, 0.50-1.30; *P* = .37). Notably, Mariya et al^27^ reported that the most common cause of death in their study cohort was active ECD (76% of 58 patients). These findings suggest that the stability of ECD drives survival outcomes in patients with IMD even in the setting of multiple intracranial lesions.

The prognosis of IMD-SE may also be influenced by ECD location, although this information was underreported among our included studies.^21^ Limited evidence on the location of extracranial metastatic sites precluded study of its impact on patient outcomes. Four studies reported OS beyond 10 years in patients with IMD-SE^32,49,60,67^ Patient characteristics for this subgroup could not be extracted and therefore, covariates associated with these extended survival times remain unknown. Although prognostic tools do not take into consideration the extracranial disease site, a thorough assessment of the location of ECD could expand treatment options and warrant more aggressive management for patients with IMD who frequently receive conservative therapy given their poor prognosis.

We also found that patients with IMD-SE demonstrated a weighted median iPFS of 13.6 months, which is longer than that of patients with IMD-PE (HR, 0.63; 95% CI, 0.52-0.76). This is in contrast to reports of iPFS in patients with brain metastases ranging from 2 to 9 months, although recent advancements in immunotherapies and targeted therapies in combination with local interventions have led to improvements in iPFS.^88^ In addition to the suggestion that ECD control may be associated with reduced intracranial disease progression, extended iPFS may be associated with delayed development of neurological symptoms and improved quality of life. This possibility is especially meaningful in the context of prolonged OS, although our analysis of iPFS is limited by differences in intracranial radiological response criteria between studies. Our median PFS estimate in patients with IMD-SE is similar to historical estimates in patients with IMD, ranging from 2 to 8.5 months, although we were unable to assess PFS differences between IMD-SE and IMD-PE.^89^

### Limitations

This systematic review and meta-analysis has several limitations. First, given the retrospective nature of most included studies, our results are limited by study quality, which we deemed to be acceptable in our analysis. Our results may also be susceptible to selection bias; our analysis, however, did not show any publication bias. Second, data on IMD-SE were frequently reported among included studies in subgroup analyses in larger studies limiting extraction of patient and disease characteristics specific to these subgroups. The absence of data on previous first-line brain-directed and systemic therapies, which may affect patient outcomes, may have introduced selection bias. The absence of reporting on intracranial disease burden may have further introduced selection bias. Moreover, among studies that reported outcomes in patients with controlled ECD, disease control was not consistently defined. This ambiguity may increase between-study heterogeneity. Third, across several studies, the number of patients with IMD-SE was not balanced with the number of patients with IMD-PE, likely a consequence of the retrospective nature of these studies. Finally, the results of our study are largely driven by the survival outcomes of patients with NSCLC, which may generate bias given that patient prognosis is associated with primary cancer and histological subtype. These limitations support the need for uniform reporting on baseline characteristics and outcomes in IMD-SE

## Conclusions

Although outcomes in patients with IMD have been historically poor, our findings suggest that IMD-SE may be associated with prolonged overall and progression-free survival. Efforts are needed to characterize this patient subgroup, predict survival, and inform treatment strategies.

## Notes

Supplement 1. **eMethods.** Supplemental Methods **eTable 1.** Search Strategy in MEDLINE (June 21, 2021) **eTable 2.** Study Definitions of Controlled ECD **eTable 3.** Characteristics of the 68 Included Studies **eTable 4.** Median OS as Reported by Studies and Derived Based on Digitized Kaplan-Meier Curves in Patients With IMD-SE vs IMD-PE **eTable 5.** Median OS as Reported by Studies and Derived Based on Digitized Kaplan-Meier Curves of Patients With BrM and Controlled vs Uncontrolled ECD **eTable 6.** iPFS of Patients With IMD-SE vs IMD-PE **eFigure 1.** OS of Patients With IMD-SE. Compared With IMD-PE in Studies That Do Not Detail Extracranial Metastases or Prior Treatment **eFigure 2.** Pooled Kaplan-Meier Survival Curves of Patients With IMD-SE **eFigure 3.** Traffic Light Plot for Risk of Bias in RCTs **eFigure 4.** Risk of Bias Summary Plot for RCTs **eFigure 5.** Traffic Light Plot for Risk of Bias in Observational Studies Comparing OS Between IMD-SE and IMD-PE **eFigure 6.** Risk of Bias Summary Plot for Observational Studies Comparing IMD-SE and IMD-PE **eFigure 7.** Traffic Light Plot for Risk of Bias in Single-Arm Observational Studies **eFigure 8.** Risk of Bias Summary Plot for Single-Arm Observational Studies **eFigure 9.** Baujat Plot for the Meta-analysis on OS of IMD-SE vs IMD-PE **eFigure 10.** Forest Plots Sorted by (A) I2 Heterogeneity and (B) Effect Size Using Leave-One-Out Meta-analysis **eFigure 11.** GOSH Plot for the Meta-analysis on OS of IMD-SE vs IMD-PE **eFigure 12.** OS of Patients With IMD-SE. Compared With IMD-PE, Excluding Mariya et al^1^ **eFigure 13.** Funnel Plot for the Meta-Analysis on OS of IMD-SE vs IMD-PE
eReferences

Supplement 2.
Data Sharing Statement
