# Supplementary material for: Association of Brain Metastases With Survival in Patients With Limited or Stable Extracranial Disease: A Systematic Review and Meta-analysis
Source: JAMA Netw Open. 2023 Feb 23;6(2):e230475. doi: 10.1001/jamanetworkopen.2023.0475 (PMC9951042; doi:10.1001/jamanetworkopen.2023.0475)
Supplement: Supplement 1. — eMethods. Supplemental Methods eTable 1. Search Strategy in MEDLINE (June 21, 2021) eTable 2. Study Definitions of Controlled ECD eTable 3. Characteristics of the 68 Included Studies eTable 4. Median OS as Reported by Studies and Derived Based on Digitized Kaplan-Meier Curves in Patients With IMD-SE vs IMD-PE eTable 5. Median OS as Reported by Studies and Derived Based on Digitized Kaplan-Meier Curves of Patients With BrM and Controlled vs Uncontrolled ECD eTable 6. iPFS of Patients With IMD-SE vs IMD-PE eFigure 1. OS of Patients With IMD-SE. Compared With IMD-PE in Studies That Do Not Detail Extracranial Metastases or Prior Treatment eFigure 2. Pooled Kaplan-Meier Survival Curves of Patients With IMD-SE eFigure 3. Traffic Light Plot for Risk of Bias in RCTs eFigure 4. Risk of Bias Summary Plot for RCTs eFigure 5. Traffic Light Plot for Risk of Bias in Observational Studies Comparing OS Between IMD-SE and IMD-PE eFigure 6. Risk of Bias Summary Plot for Observational Studies Comparing IMD-SE and IMD-PE eFigure 7. Traffic Light Plot for Risk of Bias in Single-Arm Observational Studies eFigure 8. Risk of Bias Summary Plot for Single-Arm Observational Studies eFigure 9. Baujat Plot for the Meta-analysis on OS of IMD-SE vs IMD-PE eFigure 10. Forest Plots Sorted by (A) I2 Heterogeneity and (B) Effect Size Using Leave-One-Out Meta-analysis eFigure 11. GOSH Plot for the Meta-analysis on OS of IMD-SE vs IMD-PE eFigure 12. OS of Patients With IMD-SE. Compared With IMD-PE, Excluding Mariya et al1 eFigure 13. Funnel Plot for the Meta-Analysis on OS of IMD-SE vs IMD-PE eReferences [file jamanetwopen-e230475-s001.pdf]

Supplemental Online Content

Li AY, Gaebe K, Zulfiqar A, et al. Association of brain metastases with survival in patients with limited or stable extracranial disease: a systematic review and meta-analysis. *JAMA Netw Open.* 2023;6(2):e230475. doi:10.1001/jamanetworkopen.2023.0475

eMethods. Supplemental Methods

eTable 1. Search Strategy in MEDLINE (June 21, 2021)

eTable 2. Study Definitions of Controlled ECD

eTable 3. Characteristics of the 68 Included Studies

eTable 4. Median OS as Reported by Studies and Derived Based on Digitized Kaplan-Meier Curves in Patients With IMD-SE vs IMD-PE

eTable 5. Median OS as Reported by Studies and Derived Based on Digitized Kaplan-Meier Curves of Patients With BrM and Controlled vs Uncontrolled ECD

eTable 6. iPFS of Patients With IMD-SE vs IMD-PE

eFigure 1. OS of Patients With IMD-SE. Compared With IMD-PE in Studies That Do Not Detail Extracranial Metastases or Prior Treatment

eFigure 2. Pooled Kaplan-Meier Survival Curves of Patients With IMD-SE

eFigure 3. Traffic Light Plot for Risk of Bias in RCTs

eFigure 4. Risk of Bias Summary Plot for RCTs

eFigure 5. Traffic Light Plot for Risk of Bias in Observational Studies Comparing OS Between IMD-SE and IMD-PE

eFigure 6. Risk of Bias Summary Plot for Observational Studies Comparing IMD-SE and IMD-PE

eFigure 7. Traffic Light Plot for Risk of Bias in Single-Arm Observational Studies

eFigure 8. Risk of Bias Summary Plot for Single-Arm Observational Studies

eFigure 9. Baujat Plot for the Meta-analysis on OS of IMD-SE vs IMD-PE

eFigure 10. Forest Plots Sorted by (A) I2 Heterogeneity and (B) Effect Size Using Leave-One-Out Meta-analysis

eFigure 11. GOSH Plot for the Meta-analysis on OS of IMD-SE vs IMD-PE

eFigure 12. OS of Patients With IMD-SE. Compared With IMD-PE, Excluding Mariya et al[1]

eFigure 13. Funnel Plot for the Meta-Analysis on OS of IMD-SE vs IMD-PE

eReferences

This supplemental material has been provided by the authors to give readers additional information about their work.

© 2023 Li AY et al. *JAMA Network Open.*

eMethods. Supplemental Methods

Search strategy

Grey literature sources searched in this study include Google Scholar (first 150 results), ClinicalTrials.gov, PROSPERO, Scopus, the International Clinical Trials Registry Platform of the World Health Organisation, and the websites of American Society of Clinical Oncology (ASCO), European Society of Medical Oncology (ESMO), and Society for Neuro-oncology (SNO). A sample of the search strategy performed on MEDLINE on June 21, 2021 can be found in Supplementary Table S3.

Data extraction

Four authors (K.G., A.Y.L., A.Z., G.L.) extracted pre-specified study-level data in pairs using predetermined extraction forms, including study characteristics (author, country, design, study design, follow-up), patient characteristics (age, sex, primary cancer type, performance status), and survival (overall survival and progression-free survival as medians and hazard ratios). All data items were pre-specified, collected, and reported; data that are not shown in the manuscript (treatment regimens and associated outcomes) were not reported in the included studies. Only outcomes specific to patients with IMD-SE and IMD-PE were extracted.

Data sharing statement

All study-level data used in this analysis as well as relevant software scripts (R) may be accessed at the following URL: https://www.dropbox.com/sh/ili4y7jlbxdus6q/AADfadR6rKH6k728-9yg688Za?dl=0.

© 2023 Li AY et al. *JAMA Network Open.*

eTable 1. Search strategy in MEDLINE (June 21, 2021).

#	Searches	Results
1	exp Central Nervous System Neoplasms/	190498
2	exp Cerebral Cortex/	371952
3	exp Brain/	1242152
4	exp blood-brain barrier/	29084
5	1 or 2 or 3 or 4	1388676
6	exp Neoplasm Metastasis/	210601
7	5 and 6	7199
8	exp Central Nervous System Neoplasms/sc	18431
9	((brain* or intra?cranial or cerebral or cerebrum or crani* or skull* or central nervous system or cns or leptomening* or mening* or posterior fossa or frontal lobe or parietal lobe or temporal lobe or occipital lobe or insula* or cortex or cortic* or encephal* or hippocamp* or gyrus or limbic or dentate or white matter or gr$y matter) adj3 (metasta* or (secondar* adj3 (malig* or cancer* or disease* or neoplas* or tumo?r* or carcinoma* or spread*)))).mp.	24726
10	(metasta* or (secondar* adj3 (malig* or cancer* or disease* or neoplas* or tumo?r* or carcinoma* or spread*))).mp.	640987
11	5 and 10	31603
12	7 or 8 or 9 or 11	43949
13	oligo?met*.mp.	2434
14	(secondar* adj3 (malig* or cancer* or disease* or neoplas* or tumo?r* or carcinoma* or spread*) adj5 (limit* or stable or isolat* or restrict* or control* or confin* or constrain* or local* or asymptomatic)).mp.	851
15	13 or 14	3281
16	12 and 15	298

© 2023 Li AY et al. *JAMA Network Open.*

eTable 2. Study definitions of controlled ECD.

Study	Definition of controlled ECD	Notes
Armstrong et al., 2019 [2]	information unavailable	Binary yes/no for controlled ECD
Chamberlain et al., 1996 [3]	information unavailable	Inclusion criteria of systemic disease controlled or treatable with a life expectancy $\geq$ 6 months in the absence of BrM
Gu et al., 2019 [4,*]	information unavailable	Binary yes/no for controlled ECD
Mariya et al., 2010 [1]	information unavailable	Binary yes/no for active extracranial metastatic lesions
McTyre et al., 2016 [5]	information unavailable	Binary stable/progressive for systemic disease status
Mitin et al., 2011 [6]	information unavailable	Binary yes/no for no ECD progression
Nogi et al., 2013 [7]	information unavailable	Inclusion criteria of no extracranial tumour progression within 3 months of treatment
Pessina et al., 2016 [8]	information unavailable	Inclusion criteria of controlled extracranial metastases
Pessina et al., 2017 [9,*]	information unavailable	Inclusion criteria of controlled extracranial disease
Rodrigues et al., 2011 [10]	information unavailable	Inclusion criteria for non-rapidly progressive extracranial disease OR systemic disease absent or controlled on treatment

BrM, brain metastases; CI, confidence interval; ECD, extracranial disease; IMD-SE, intracranial metastatic disease in the setting of stable ECD.
[*]Study reports two separate cohorts of patients, including one cohort of patients with IMD and stable or controlled ECD without further detailing extracranial metastases or prior treatment, as well as one cohort of patient that explicitly met our criteria for IMD-SE.

© 2023 Li AY et al. *JAMA Network Open.*

eTable 3. Characteristics of the 68 included studies.

Study	Publication Type	Study Design	Primary Cancer	IMD-SE Criteria	Median Follow-up (months)	IMD-SE (*n*)	IMD-PE (*n*)
Alhalabi et al., 2021 [11]	Full text	RCS	BC	≤ 2 EC sites	–	74	–
Andrews et al., 2004 [12]	Full text	RCT	Mixed	≤ 2 EC sites	–	291	–
Aoyama et al., 2003 [13]	Full text	RCS	Mixed	≤ 2 EC sites	6.3	34	53
Armstrong et al., 2019 [2]	Full text	RCS	BC	Controlled ECD	10.33	35	16
Bai et al., 2016 [14]	Full text	RCS	NSCLC	≤ 2 EC sites	–	76	–
Balducci et al., 2015 [15]	Full text	RCS	Mixed	≤ 2 EC sites	102	47	–
Bates et al., 2015 [16]	Full text	RCS	Melanoma	≤ 2 EC sites	–	10	63
Bilani et al., 2020 [17]	Full text	RCS	BC	≤ 2 EC sites	–	805	–
Bodor et al., 2019 [18]	Full text	RCS	NSCLC	≤ 2 EC sites	–	23	–
Buglione et al., 2020 [19]	Full text	RCS	NSCLC	≤ 2 EC sites	18	108	–
Chamberlain et al., 1996 [3]	Full text	PCS	Mixed	Controlled ECD	–	20	30
Chen et al., 2021 [20]	Full text	RCS	NSCLC	≤ 2 EC sites	49	125	127
Cheufou et al., 2014 [21]	Full text	RCS	NSCLC	≤ 2 EC sites	17.3	37	–
Churilla et al., 2017 [22]	Full text	RCT	Mixed	≤ 2 EC sites	26	329	
Collaud et al., 2012 [23]	Full text	RCS	NSCLC	≤ 2 EC sites	–	19	–
Congedo et al., 2012 [24]	Full text	RCS	NSCLC	≤ 2 EC sites	28	39	–
D'Agostino et al., 2011 [25]	Full text	RCS	Mixed	≤ 2 EC sites	95	97	
Della Seta et al., 2019 [26]	Full text	RCS	Mixed	≤ 2 EC sites	–	13	35
Endo et al., 2014 [27]	Full text	PCS	NSCLC	≤ 2 EC sites	54.4	17	–
Ferro et al., 2016 [28]	Full text	PCS	Mixed	≤ 2 EC sites	76	30	
Frost et al., 2018 [29]	Full text	RCS	Lung	≤ 2 EC sites	Intervention: 32.2<br>Control: 18.8	80	–
Gauvin et al., 2021 [30]	Full text	RCS	NSCLC	≤ 2 EC sites	13	50	–
Gorovets et al., 2014 [31,*]	Abstract	RCS	Mixed	≤ 2 EC sites	9.4	–	–
Gorovets et al., 2015 [32,*]	Full text	RCS	Mixed	≤ 2 EC sites	–	78	65
Gorovets et al., 2016 [33,*]	Full text	RCS	Mixed	≤ 2 EC sites	72.7	255	297
Gray et al., 2014 [34]	Full text	RCS	NSCLC	≤ 2 EC sites	31.9	66	–
Griffioen et al., 2013 [35]	Full text	RCS	NSCLC	≤ 2 EC sites	26.1	36	–
Gu et al., 2019 [4]	Full text	RCS	Mixed	≤ 2 EC sites	48.5	70	91
Guo et al., 2014 [36]	Abstract	RCS	NSCLC	≤ 2 EC sites	17.2	53	–
Harat et al., 2020 [37]	Full text	RCS	Mixed	≤ 2 EC sites	9.5	82	–
Hirschmann et al., 2018 [38]	Abstract	RCS	NSCLC	≤ 2 EC sites	79.5	–	–
Inoue et al., 2010 [39]	Full text	RCS	Mixed	≤ 2 EC sites	20	24	–
Kaba et al., 2021 [40]	Full text	RCS	NSCLC	≤ 2 EC sites	mean ± SD (min-max): 25.71 ± 23.47 (4-92)	28	–
Karlovits et al., 2009 [41]	Full text	RCS	Mixed	≤ 2 EC sites	13	27	25
Kocher et al., 2010 [42]	Full text	RCT	Mixed	≤ 2 EC sites	49	359	–
Loi et al., 2019 [43]	Full text	RCS	NSCLC	≤ 2 EC sites	–	42	–
Lopez Guerra et al., 2012 [44]	Full text	RCS	NSCLC	≤ 2 EC sites	–	27	–
Macchia et al., 2015 [45]	Abstract	PCS	–	≤ 2 EC sites	–	27	–
Mariya et al., 2010 [1]	Full text	RCS	NSCLC	Controlled ECD	8.5	21	63
McTyre et al., 2016 [5]	Full text	RCS	Mixed	Controlled ECD	53.9	399	264
Mitchell et al., 2020 [46]	Full text	RCS	NSCLC	≤ 2 EC sites	52.3	86	–
Mitin et al., 2011 [6,*]	Abstract	RCS	–	Controlled ECD	–	–	–
Mitin et al., 2013 [47,*]	Full text	RCS	Mixed	≤ 2 EC sites	16.2	46	123
Naqash et al., 2019 [48]	Abstract	RCS	NSCLC	≤ 2 EC sites	–	42	–
Navarria et al., 2019 [49]	Abstract	PCS	Mixed	≤ 2 EC sites	25	135	–
Nieder et al., 2020 [50,*]	Full text	RCS	Mixed	≤ 2 EC sites	15	89	–
Nieder et al., 2020 [51,*]	Full text	RCS	Mixed	≤ 2 EC sites	25	198	–
Niibe et al., 2016 [52]	Full text	RCS	NSCLC	≤ 2 EC sites	–	61	–
Nikitas et al., 2020 [53]	Full text	RCS	NSCLC	≤ 2 EC sites	9	6	–
Nogi et al., 2013 [7]	Full text	RCS	Mixed	Controlled ECD	–	59	159
Pessina et al., 2016 [8,*]	Full text	RCS	Mixed	Controlled ECD	24	69	–
Pessina et al., 2017 [9,*]	Full text	RCS	NSCLC	≤ 2 EC sites	14.8	101	55
Pikin et al., 2011 [54,*]	Abstract	RCS	NSCLC	≤ 2 EC sites	–	32	–
Pikin et al., 2017 [55,*]	Abstract	RCS	NSCLC	≤ 2 EC sites	52	82	–
Raez et al., 2019 [56]	Abstract	RCS	NSCLC	≤ 2 EC sites	–	45	–
Rodrigues et al., 2011 [10]	Full text	RCS	Mixed	Controlled ECD	4.7	–	–
Rogers et al., 2006 [57]	Full text	PCS	Mixed	≤ 2 EC sites	–	31	–

© 2023 Li AY et al. *JAMA Network Open.*

Study	**Publication Type**	**Study Design**	**Primary Cancer**	**IMD-SE Criteria**	**Median Follow-up (months)**	**IMD-SE (*n*)**	**IMD-PE (*n*)**
Salvador Coloma et al., 2018 [58]	Abstract	RCS	NSCLC	≤ 2 EC sites	–	67	–
Sato et al., 2018 [59]	Full text	RCS	NSCLC	≤ 2 EC sites	16	19	–
Shibata et al., 2019 [60]	Full text	RCS	SCLC	≤ 2 EC sites	–	11	–
Shirasawa et al., 2019 [61]	Full text	RCS	SCLC	≤ 2 EC sites	–	6	–
Song et al., 2020 [62]	Full text	RCS	NSCLC	≤ 2 EC sites	11	5	–
Suzuki et al., 2021 [63]	Full text	PCS	NSCLC	≤ 2 EC sites	63	18	–
Wang et al., 2018 [64]	Full text	RCS	NSCLC	≤ 2 EC sites	Range: 12-72	74	–
Xu et al., 2018 [65]	Abstract	RCS	NSCLC	≤ 2 EC sites	–	41	–
Yamaguchi et al., 2017 [66]	Full text	RCS	NSCLC	≤ 2 EC sites	37.8	13	–
Yegya-Raman et al., 2019 [67]	Full text	RCS	NSCLC	≤ 2 EC sites	54.9	18	–
Zhang et al., 2019 [68]	Full text	RCS	NSCLC	≤ 2 EC sites	20.87	18	–

BC, breast cancer; EC, extracranial; ECD, extracranial disease; IMD-PE, intracranial metastatic disease in the setting of progressive extracranial disease; IMD-SE, intracranial metastatic disease in the setting of stable extracranial disease; NSCLC, non-small cell lung cancer; PCS, prospective cohort study; RCS, retrospective cohort study; RCT, randomized controlled trial; SCLC, small cell lung cancer; SD, standard deviation; –, information unavailable.

[*] Several studies were published by the same primary author across different years. All articles were screened to ensure minimal-to-overlap between study cohorts. Gorovets et al., 2014[31] and 2015[32] were not included in our analysis as they did not report on our pre-specified primary/secondary outcomes. Mitin et al., 2011[6] and 2014[47] reported on different outcome measures that were incorporated into our meta-analysis and pooled survival analysis. Nieder et al., 2020[50] and 2020[51] reported on two different cohorts of patients. Pessina et al., 2016[8] and 2017[9] reported on two different cohorts of patients. Pikin et al. 2011[54] and 2017[55] report on cohorts of patients with different baseline characteristics.

© 2023 Li AY et al. *JAMA Network Open.*

eTable 4. Median OS as reported by studies and derived based on digitized Kaplan-Meier curves in patients with IMD-SE versus IMD-PE. All studies listed were included in the primary meta-analysis comparing OS of IMD-SE with IMD-PE except Mitin et al., 2013 [47] and Hirschmann et al., 2018 [38] due to insufficient data.

**Study**	**IMD-SE**					**IMD-PE**					**HR**			
	**No. patients**	**Median OS Reported**		**Median OS Derived**		**No. patients**	**Median OS Reported**		**Median OS Derived**		**HR Reported**		**HR Derived**	
		Months	95% CI	Months	95% CI		Months	95% CI	Months	95% CI	HR	95% CI	HR	95% CI
Gu et al., 2019 [4]	70	24	–	–		91	13	–	–		0.61	0.4-0.9	–	–
Armstrong et al., 2019 [2]	35	21.9	12.6-31.1	21	12-42	16	7.3	4-10.6	7.1	5.1-	–	–	0.33	0.1-0.7
Bates et al., 2015 [16,*]	10	15.2	–	–		63	2.4	–	–		0.34	0.2-0.7	–	–
Chen et al., 2021 [20]	125	–	–	–		127	–	–	–		0.66	0.5-0.9	–	–
Mitin et al., 2013 [47]	46	21.7	–	–		123	10.3	–	–		–	–	–	–
Mariya et al., 2010 [1]	21	32	–	32.4	14.9-	63	7	–	7.5	5.6-9.5	–	–	0.18	0.1-0.3
Mitin et al., 2011 [6,*]	–	–	–	–		–	–	–	–		0.64	0.4-1.0	–	–
Hirschmann et al., 2018 [38]	–	–	–	–		–	–	–	–		–	–	–	–
Karlovits et al., 2009 [41]	27	22	–	22.3	16.3-	25	13	–	12.8	8.3-17.3	–	–	0.42	0.2-0.8
Della Seta et al., 2019 [26,*]	13	–	–	–		35	–	–	–		0.56	0.3-1.2	–	–
Pessina et al., 2017 [9]	101	18.6	13.6-23.6	–		55	12.1	9.7-14.5	–		0.91	0.5-2.3	–	–
Rodrigues et al., 2011 [10]	–	–	–	–		–	–	–	–		0.81	0.5-1.3	–	–

In studies where a univariable HR was not reported, published Kaplan-Meier curves were digitized, from which median OS and HR were derived using the method by Guyot et al. [69]; CI, confidence interval; HR, hazard ratio; IMD-PE, intracranial metastatic disease in the setting of progressive extracranial disease; IMD-SE, intracranial metastatic disease in the setting of stable extracranial disease; OS, overall survival; –, information unavailable.

[*] Median follow-up information was not reported in these studies.

© 2023 Li AY et al. *JAMA Network Open.*

eTable 5. Median OS as reported by studies and derived based on digitized Kaplan-Meier curves of patients with BrM and controlled versus uncontrolled ECD.

**Study**	**Controlled Extracranial Disease (IMD-SE)**					**Uncontrolled Extracranial Disease (IMD-PE)**					**HR**			
	**No. patients**	**Median OS Reported**		**Median OS Derived**		**No. patients**	**Median OS Reported**		**Median OS Derived**		**HR Reported**		**HR Derived**	
		mo	95% CI	mo	95% CI		mo	95% CI	mo	95% CI	HR	95% CI	HR	95% CI
Gu et al., 2019 [4,*]	–	24	–	–	–	–	13.5	–	–	–	0.60	0.4-0.9	–	–
Armstrong et al., 2019 [2]	35	21.9	12.6-31.1	21	12-42	16	7.3	4-10.6	7.1	5.1-	–	–	0.33	0.1-0.7
Mariya et al., 2010 [1]	21	32	–	32.4	14.9-	63	7	–	7.52	5.6-9.5	–	–	0.18	0.1-0.3
Mitin et al., 2011 [6,**]	–	–	–	–	–	–	–	–	–	–	0.62	0.4-1.0	–	–
Rodrigues et al., 2011 [10]	–	–	–	–	–	–	–	–	–	–	0.81	0.5-1.3	–	–

In studies where a univariable hazard ratio (HR) was not reported, published Kaplan-Meier curves were digitized, from which median overall survival (OS) and HR were derived using the method by Guyot et al. [69]

BrM, brain metastases; CI, confidence interval; ECD, extracranial disease; HR, hazard ratio; IMD-PE, intracranial metastatic disease in the setting of progressive extracranial disease; IMD-SE, intracranial metastatic disease in the setting of stable extracranial disease; mo, months; OS, overall survival; –, information unavailable.

[*] Median OS and HRs from the subgroup of patients with BrM and controlled versus uncontrolled ECD were derived for meta-analysis.

[**] Median follow-up information was not reported in this study.

© 2023 Li AY et al. *JAMA Network Open.*

eTable 6. Reported iPFS of patients with IMD-SE versus IMD-PE.

Study	IMD-SE			IMD-PE			HR		*P*-value
	**No. patients**	**Median iPFS**		**No. patients**	**Median iPFS**		HR	95% CI	
		mo	95% CI		mo	95% CI			
Della Seta et al., 2019 [26,*]	13	–	–	35	–	–	0.78	0.3-2.2	0.64
Rodrigues et al., 2011 [10]	–	–	–	–	–	–	0.74	0.3-2.0	0.55
Gorovets et al., 2016 [33]	255	13.6	10.2-15	297	5.5	4.9-6.6	0.64	0.5-0.8	< 0.001
Bates et al., 2015 [16,*]	10	5	–	63	1.9	–	0.41	0.2-0.9	0.021

CI, confidence interval; HR, hazard ratio; IMD-PE, intracranial metastatic disease in the setting of progressive extracranial disease; IMD-SE, intracranial metastatic disease in the setting of stable extracranial disease; iPFS, intracranial progression-free survival; mo, months; –, information unavailable.

[*] Median follow-up information was not reported in these studies.

© 2023 Li AY et al. *JAMA Network Open.*

Author	Primary cancer	No. patients (IMD-SE)	No. patients (IMD-PE)	NOS Score	AHRQ	HR	95% CI	Weight
Mariya et al,[1] 2010	NSCLC	21	63	8	Good quality	0.18	[0.09; 0.35]	18.1%
Armstrong et al,[2] 2019	BC	35	16	8	Good quality	0.33	[0.15; 0.73]	16.3%
Gu et al,[4] 2019	Mixed	NR	NR	7	Good quality	0.60	[0.40; 0.89]	22.5%
Mitin et al,[9] 2011	NR	NR	NR	7	Poor quality	0.64	[0.41; 1.00]	21.8%
Rodrigues et al,[10] 2011	Mixed	NR	NR	6	Poor quality	0.81	[0.50; 1.30]	21.4%
**Random effects model**						**0.47**	**[0.28; 0.80]**	**100.0%**
**Prediction interval**							**[0.07; 3.16]**	

Heterogeneity: $I^2 = 74\%$, $\tau^2 = 0.2854$, $p < 0.01$

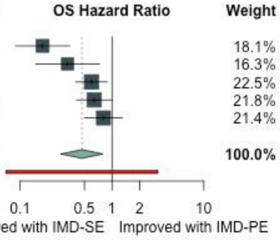

eFigure 1. Random-effects meta-analysis of the primary outcome of OS in patients with IMD-SE versus IMD-PE in studies that do not detail extracranial metastases or prior treatment. AHRQ, Agency for Healthcare Research and Quality; BC, breast cancer; IMD-PE, intracranial metastatic disease in the setting of progressive extracranial disease; IMD-SE, intracranial metastatic disease in the setting of stable extracranial disease; NOS, Newcastle-Ottawa Scale; NR, not reported; NSCLC, non-small cell lung cancer; OS, overall survival. The number of patients with IMD-SE and IMD-PE were not reported in three studies and were not included in the total number of patients.[4,6,10]

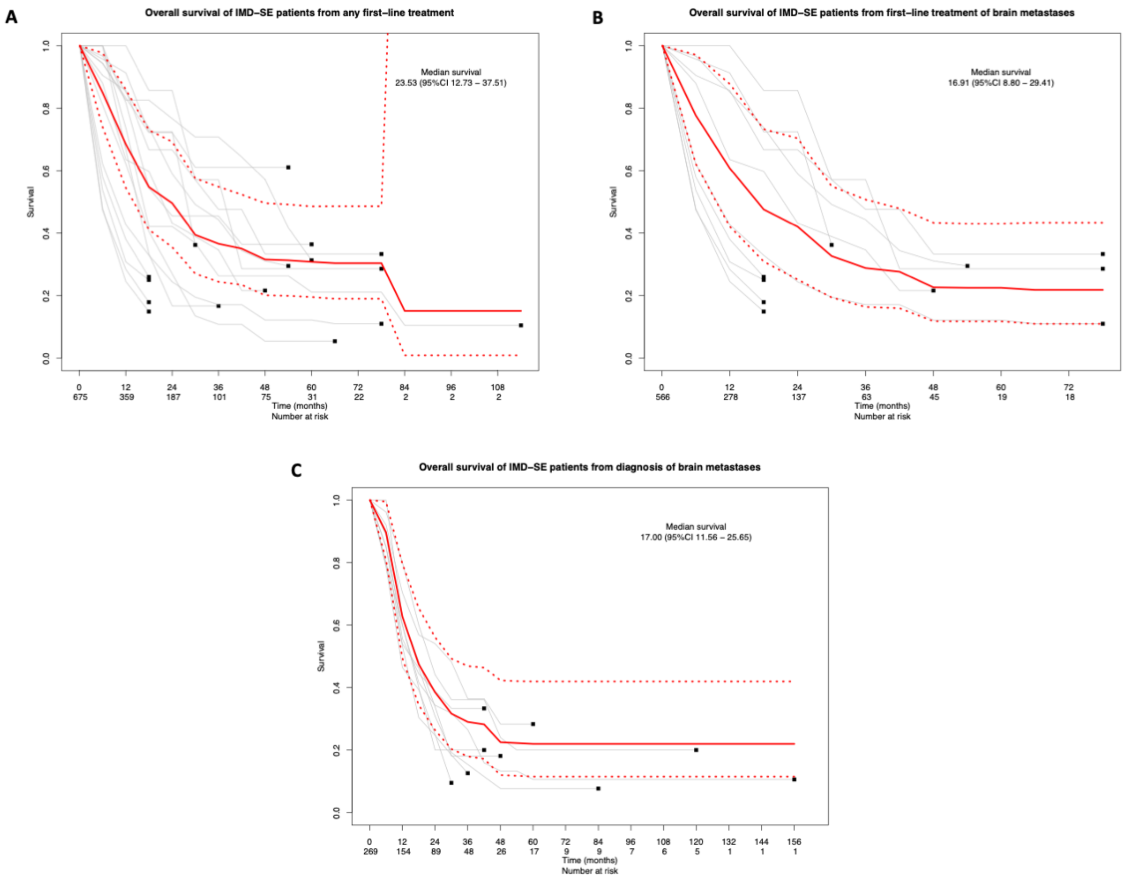

eFigure 2. Summary OS curves of patients with IMD-SE. Overall survival (OS) from (A) any first-line treatment, including from treatment of primary cancer and/or brain metastases, (B) treatment of brain metastases only, and (C) diagnosis of brain metastases. Grey lines represent OS curves for individual studies. The red solid lines represent the summary survival curves, and the red dashed lines represent the 95% confidence interval. IMD-SE, intracranial metastatic disease in the setting of stable extracranial disease.

© 2023 Li AY et al. *JAMA Network Open.*

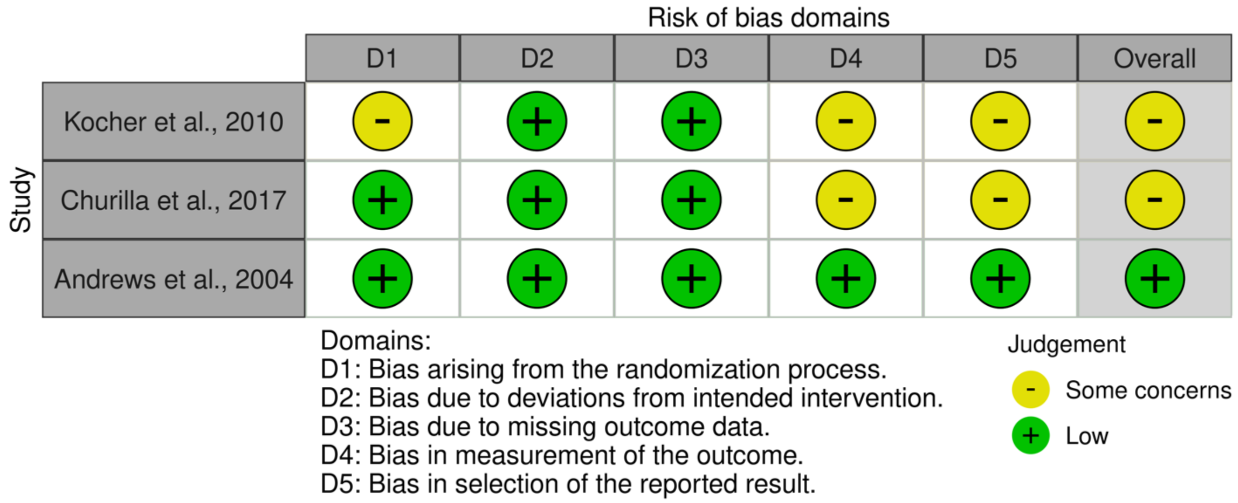

eFigure 3. Traffic light plot for risk of bias in RCTs.

© 2023 Li AY et al. *JAMA Network Open.*

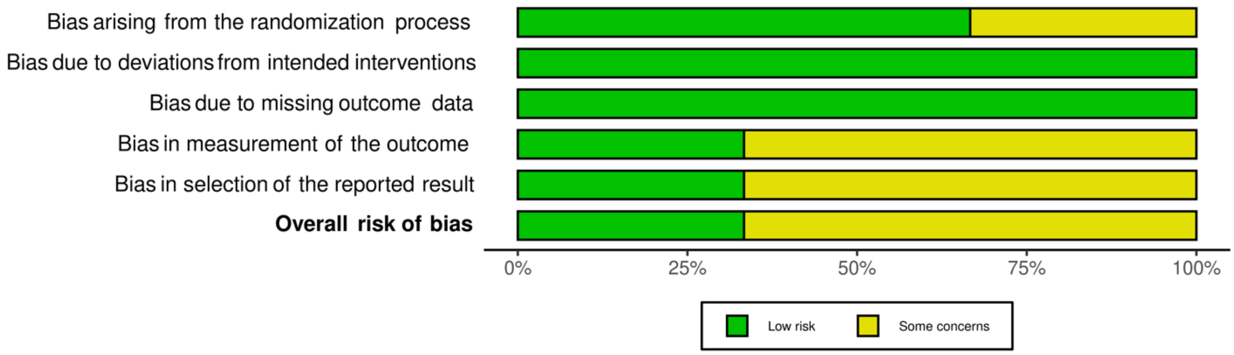

eFigure 4. Risk of bias summary plot for RCTs.

© 2023 Li AY et al. *JAMA Network Open.*

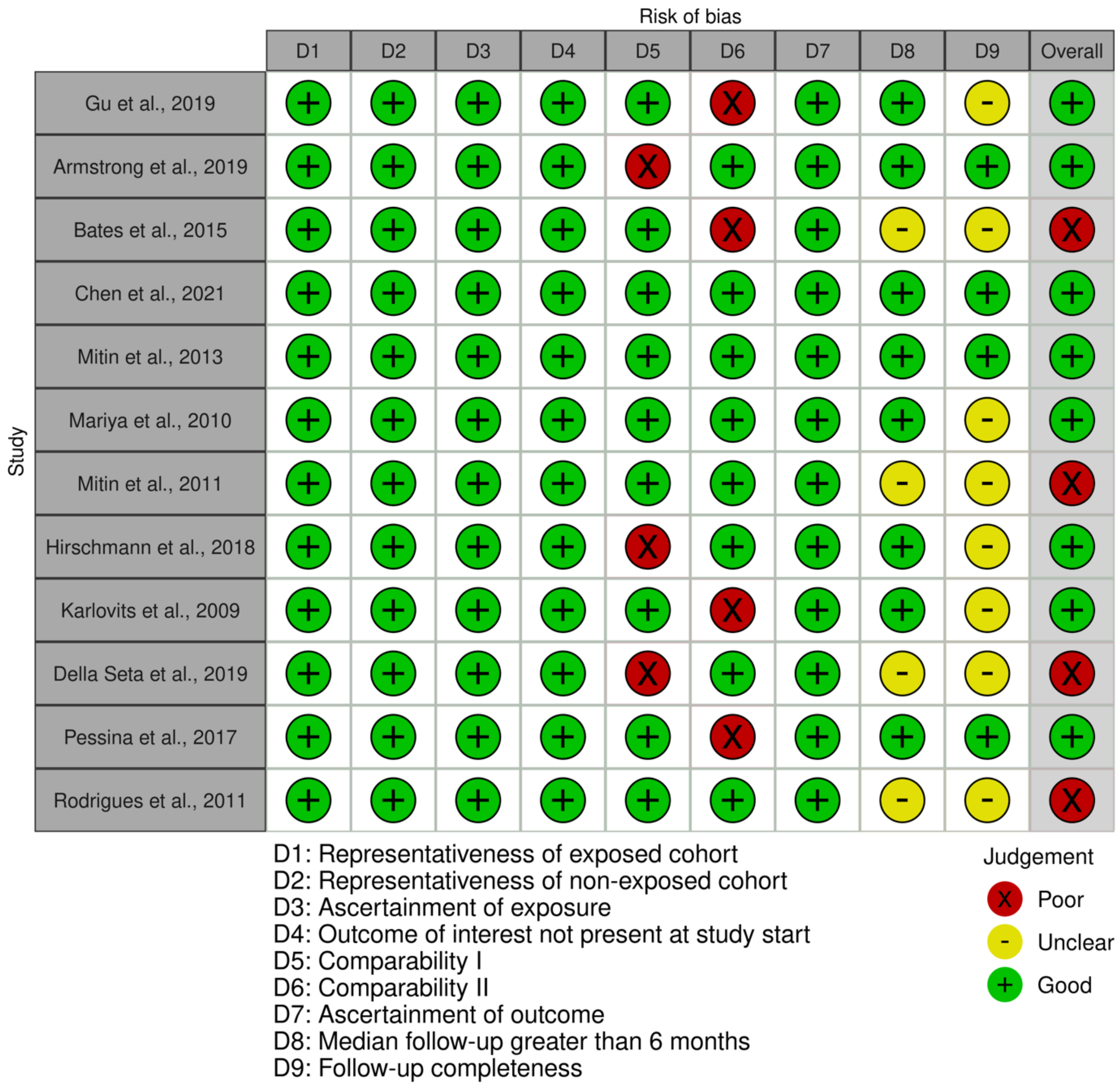

eFigure 5. Traffic light plot for risk of bias in observational studies comparing OS between IMD-SE and IMD-PE. IMD-PE, intracranial metastatic disease in the setting of progressive extracranial disease; IMD-SE, intracranial metastatic disease in the setting of stable extracranial disease; OS, overall survival.

© 2023 Li AY et al. *JAMA Network Open.*

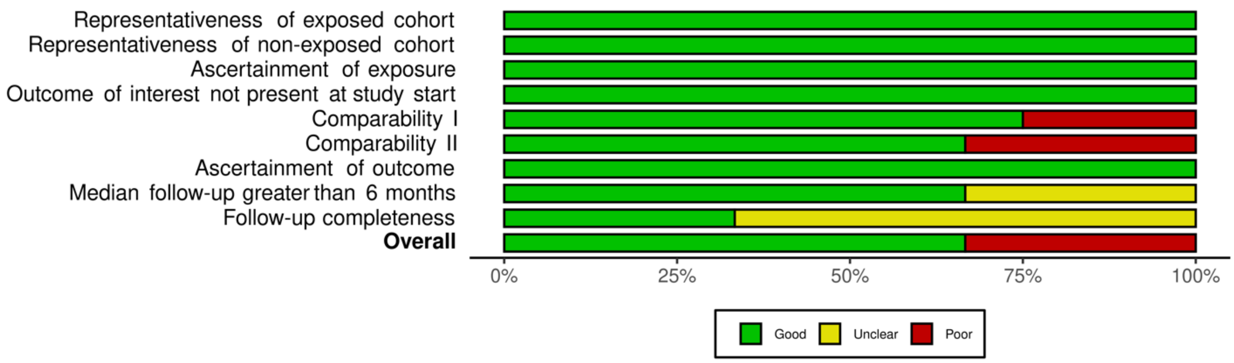

eFigure 6. Risk of bias summary plot for observational studies comparing IMD-SE and IMD-PE. IMD-PE, intracranial metastatic disease in the setting of progressive extracranial disease; IMD-SE, intracranial metastatic disease in the setting of stable extracranial disease.

© 2023 Li AY et al. *JAMA Network Open.*

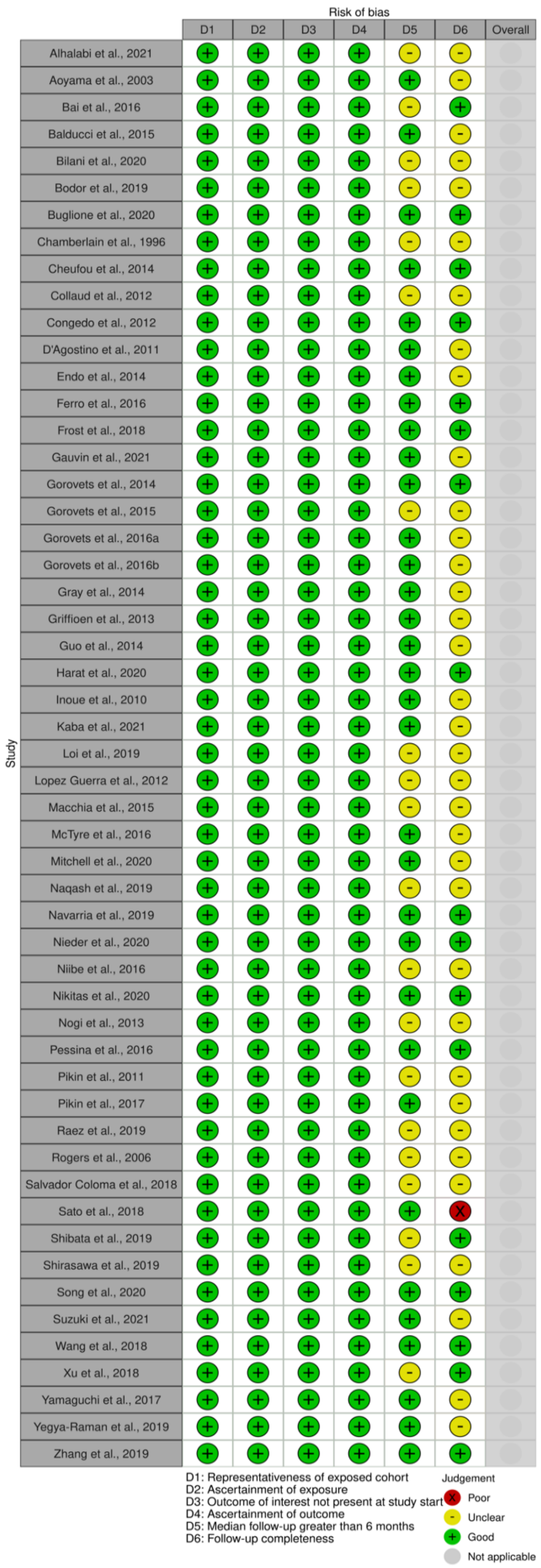

eFigure 7. Traffic light plot for risk of bias in single-arm observational studies.

© 2023 Li AY et al. *JAMA Network Open.*

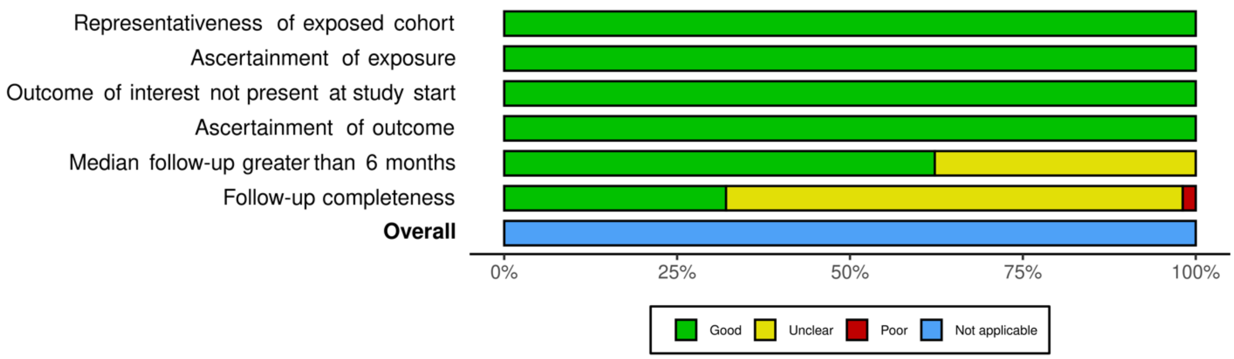

eFigure 8. Risk of bias summary plot for single-arm observational studies.

© 2023 Li AY et al. *JAMA Network Open.*

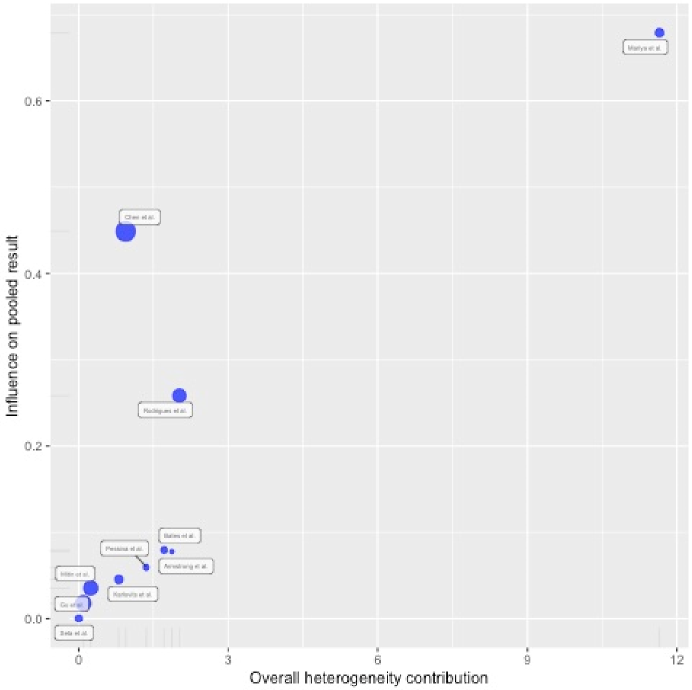

eFigure 9. Baujat plot for the meta-analysis on OS of IMD-SE versus IMD-PE. IMD-PE, intracranial metastatic disease in the setting of progressive extracranial disease; IMD-SE, intracranial metastatic disease in the setting of stable extracranial disease; OS, overall survival.

© 2023 Li AY et al. *JAMA Network Open.*

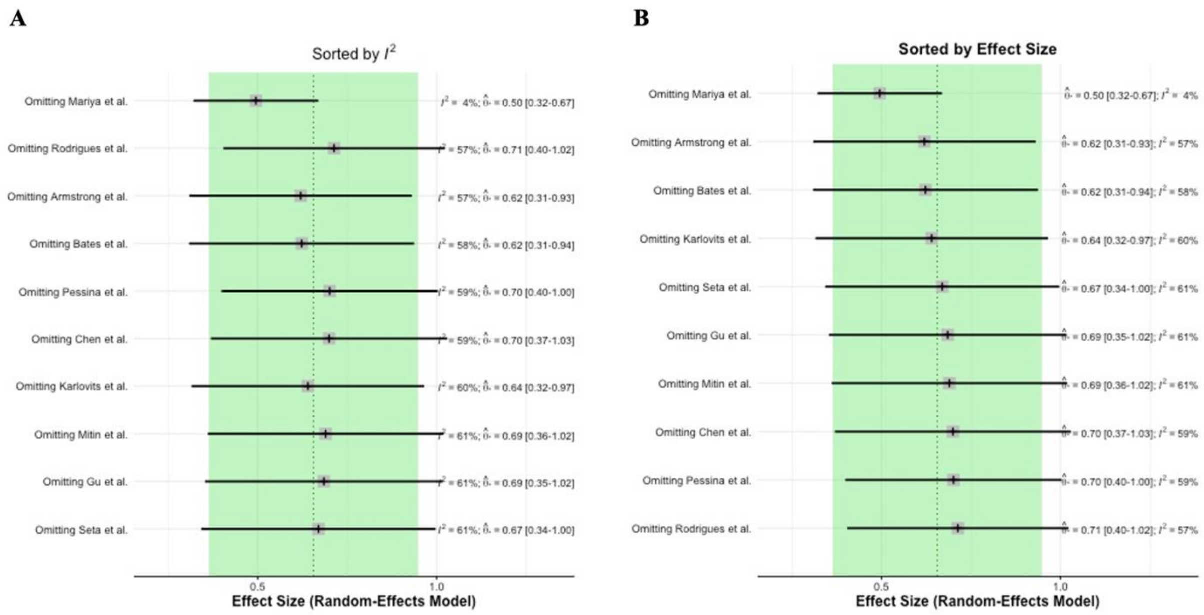

eFigure 10. Forest plots sorted by (A) I^2 heterogeneity and (B) effect size using leave-one-out meta-analysis.

© 2023 Li AY et al. *JAMA Network Open.*

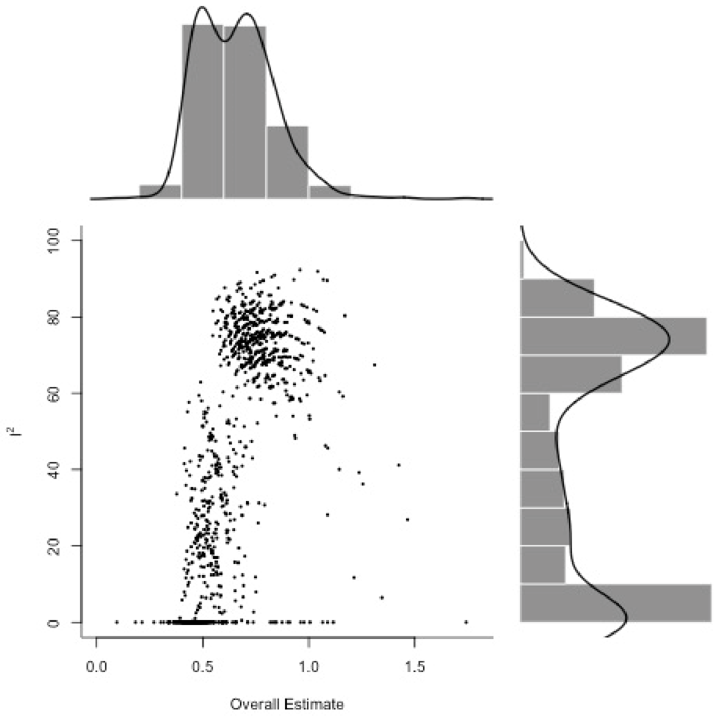

eFigure 11. GOSH plot for the meta-analysis on OS of IMD-SE versus IMD-PE. GOSH, graphic display of heterogeneity; IMD-PE, intracranial metastatic disease in the setting of progressive extracranial disease; IMD-SE, intracranial metastatic disease in the setting of stable extracranial disease; OS, overall survival.

© 2023 Li AY et al. *JAMA Network Open.*

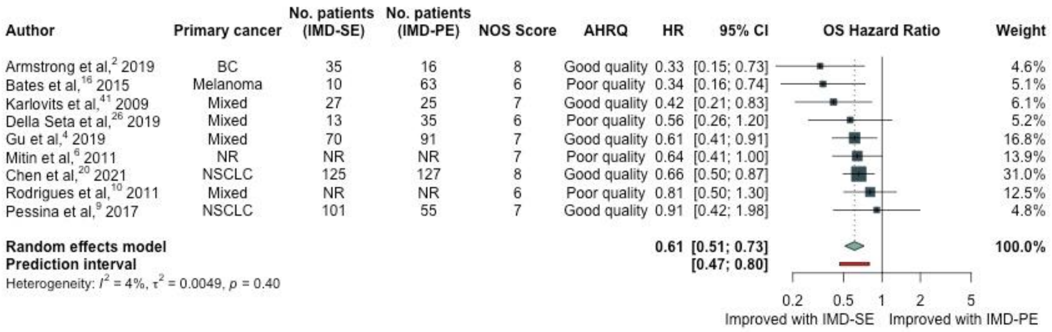

eFigure 12. Random effects meta-analysis of the primary outcome of OS in patients with IMD-SE compared to IMD-PE, excluding cohorts reported in Mariya et al. [1] AHRQ, Agency for Healthcare Research and Quality; BC, breast cancer; CI, confidence interval; HR, hazard ratio; IMD-PE, intracranial metastatic disease in the context of progressive extracranial disease; IMD-SE, intracranial metastatic disease in the context of stable extracranial disease; NOS, Newcastle-Ottawa Scale; NR, not reported; NSCLC, non-small cell lung cancer; OS, overall survival. The number of patients with IMD-SE and IMD-PE were not reported in two studies and were not included in the total number of patients.[6,10]

© 2023 Li AY et al. *JAMA Network Open.*

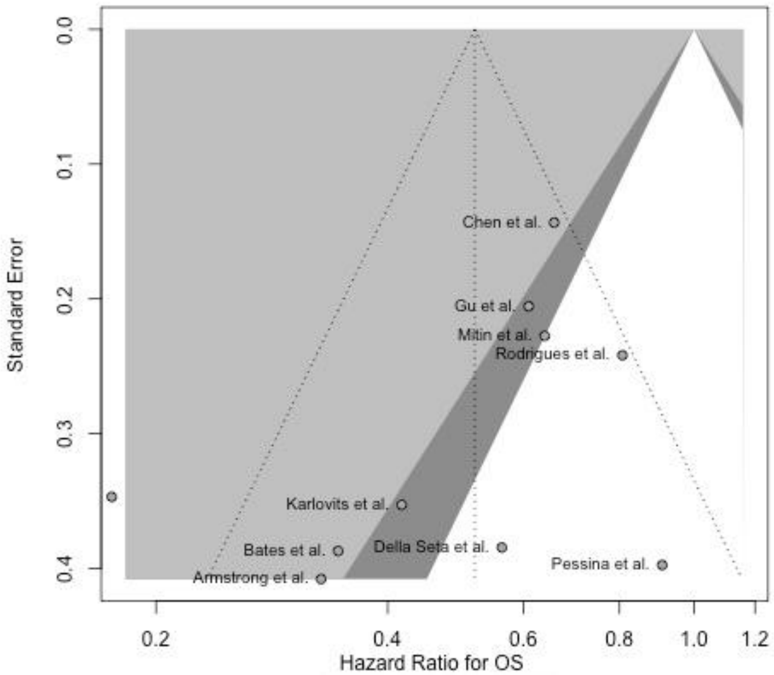

eFigure 13. Funnel plot for the meta-analysis on OS of IMD-SE versus IMD-PE. IMD-PE, intracranial metastatic disease in the setting of progressive extracranial disease; IMD-SE, intracranial metastatic disease in the setting of stable extracranial disease; OS, overall survival.

© 2023 Li AY et al. *JAMA Network Open.*

eREFERENCES

1. Mariya Y, Sekizawa G, Matsuoka Y, Seki H, Sugawara T. Outcome of stereotactic radiosurgery for patients with non-small cell lung cancer metastatic to the brain. *Journal of radiation research.* 2010; 51(3):333-342.

2. Armstrong K, Ward J, Dunne M, et al. Linac-based radiosurgery for patients with brain oligometastases from a breast primary, in the trastuzumab era-impact of tumor phenotype and prescribed SRS dose. *Frontiers in Oncology.* 2019; 9(MAY).

3. Chamberlain M, Kormanik P, Barba D, Fuller B, Smith D, Shea W. Stereotactic radiosurgery for metastatic brain tumors. *International journal of oncology.* 1996; 8(3):617-624.

4. Gu L, Qing S, Zhu X, et al. Stereotactic Radiation Therapy (SRT) for Brain Metastases of Multiple Primary Tumors: A Single Institution Retrospective Analysis. *Frontiers in Oncology.* 2019; 9.

5. McTyre ER, Johnson AG, Ruiz J, et al. Predictors of neurologic and nonneurologic death in patients with brain metastasis initially treated with upfront stereotactic radiosurgery without whole-brain radiation therapy. *Neuro-Oncology.* 2016; 19(4):558-566.

6. Mitin T, Floyd SR, Kasper EM, Mahadevan A. The effect of systemic disease status on overall survival and CNS disease control in patients with newly diagnosed brain oligometastases and treated with stereotactic radiosurgery alone. *International Journal of Radiation Oncology Biology Physics.* 2011; 81(2 SUPPL. 1):S647.

7. Nogi S, Nakayama H, Tajima Y, et al. Significance of the number of brain metastases for identifying patients who don't need whole brain radiotherapy: implication as oligometastases of the brain. *Journal of radiosurgery and SBRT.* 2013; 2(2):119-126.

8. Pessina F, Navarria P, Cozzi L, et al. Outcome evaluation of oligometastatic patients treated with surgical resection followed by Hypofractionated Stereotactic Radiosurgery (HSRS) on the tumor bed, for single, large brain metastases. *PLoS ONE.* 2016; 11(6).

9. Pessina F, Navarria P, Cozzi L, et al. Outcome appraisal of patients with limited brain metastases (BMs) from non small cell lung cancer (NSCLC) treated with different local therapeutic strategies: A single institute evaluation. *British Journal of Radiology.* 2017; 90(1072).

10. Rodrigues G, Eppinga W, Lagerwaard F, et al. A pooled analysis of arc-based image-guided simultaneous integrated boost radiation therapy for oligometastatic brain metastases. *Radiotherapy and oncology.* 2011; 102(2):180-186.

11. Alhalabi O, Soomro Z, Sun R, et al. Outcomes of changing systemic therapy in patients with relapsed breast cancer and 1 to 3 brain metastases. *npj Breast Cancer.* 2021; 7(1).

12. Andrews DW, Scott CB, Sperduto PW, et al. Whole brain radiation therapy with or without stereotactic radiosurgery boost for patients with one to three brain metastases: phase III results of the RTOG 9508 randomised trial. *The Lancet (British edition).* 2004; 363(9422):1665-1672.

13. Aoyama H, Shirato H, Onimaru R, et al. Hypofractionated stereotactic radiotherapy alone without whole-brain irradiation for patients with solitary and oligo brain metastasis using noninvasive fixation of the skull. *International Journal of Radiation Oncology Biology Physics.* 2003; 56(3):793-800.

14. Bai H, Xu J, Yang H, et al. Survival prognostic factors for patients with synchronous brain oligometastatic non-small-cell lung carcinoma receiving local therapy. *OncoTargets and Therapy.* 2016; 9:4207-4213.

© 2023 Li AY et al. *JAMA Network Open.*

15. Balducci M, Autorino R, Chiesa S, et al. Radiosurgery or Fractionated Stereotactic Radiotherapy plus Whole-brain Radioherapy in Brain Oligometastases: A Long-term Analysis. *Anticancer Research.* 2015; 35(5):3055.
16. Bates JE, Youn P, Usuki KY, et al. Brain metastasis from melanoma: the prognostic value of varying sites of extracranial disease. *Journal of Neuro-Oncology.* 2015; 125(2):411-418.
17. Bilani N, Elson L, Liang H, Elimimian EB, Nahleh Z. Effect of Surgery at Primary and Metastatic Sites in Patients with Stage IV Breast Cancer. *Clinical Breast Cancer.* 2020.
18. Bodor JN, Edelman MJ, Feliciano JL. Outcomes of patients with disease recurrence after treatment for locally advanced non-small cell lung cancer detected by routine follow-up CT scans versus a symptom driven evaluation. *Lung Cancer.* 2019; 135:16-20.
19. Buglione M, Jereczek-Fossa BA, Bonù ML, et al. Radiosurgery and fractionated stereotactic radiotherapy in oligometastatic/oligoprogressive non-small cell lung cancer patients: Results of a multi-institutional series of 198 patients treated with “curative” intent. *Lung Cancer.* 2020; 141:1-8.
20. Chen XR, Hou X, Li DL, et al. Management of Non–Small-Cell Lung Cancer Patients Initially Diagnosed With 1 to 3 Synchronous Brain-Only Metastases: A Retrospective Study. *Clinical Lung Cancer.* 2021; 22(1):e25-e34.
21. Cheufou DH, Welter S, Chalvatzoulis E, Christof D, Theegarten D, Stamatis G. Surgery of primary lung cancer with oligometastatic m1b synchronous single brain metastasis: analysis of 37 cases. *The Thoracic and cardiovascular surgeon.* 2014; 62(7):612-615.
22. Churilla TM, Handorf E, Collette S, et al. Whole brain radiotherapy after stereotactic radiosurgery or surgical resection among patients with one to three brain metastases and favorable prognoses: a secondary analysis of EORTC 22952-26001. *Annals of oncology.* 2017; 28(10):2588-2594.
23. Collaud S, Inci I, Schneiter D, et al. Survival of patients treated surgically for synchronous single-organ metastatic NSCLC and advanced pathologic TN stage. *Lung Cancer.* 2012; 78(3):234-238.
24. Congedo MT, Cesario A, Lococo F, et al. Surgery for oligometastatic non-small cell lung cancer: Long-term results from a single center experience. *Journal of Thoracic and Cardiovascular Surgery.* 2012; 144(2):444-452.
25. D'Agostino GR, Autorino R, Pompucci A, et al. Whole-brain radiotherapy combined with surgery or stereotactic radiotherapy in patients with brain oligometastases: Long-term analysis. *Strahlentherapie und Onkologie.* 2011; 187(7):421-425.
26. Della Seta M, Collettini F, Chapiro J, et al. A 3D quantitative imaging biomarker in pre-treatment MRI predicts overall survival after stereotactic radiation therapy of patients with a singular brain metastasis. *Acta radiologica (1987).* 2019; 60(11):1496-1503.
27. Endo C, Hasumi T, Matsumura Y, et al. A prospective study of surgical procedures for patients with oligometastatic non-small cell lung cancer. *The Annals of thoracic surgery.* 2014; 98(1):258-264.
28. Ferro MMD, Chiesa SMD, Macchia GMD, et al. Intensity Modulated Radiation Therapy With Simultaneous Integrated Boost in Patients With Brain Oligometastases: A Phase 1 Study (ISIDE-BM-1). *International journal of radiation oncology, biology, physics.* 2016; 97(1):82-90.

© 2023 Li AY et al. *JAMA Network Open.*

29. Frost N, Raspe M, Ruwwe-Glosenkamp C, et al. Local ablative treatment for synchronous single organ oligometastatic lung cancer-A propensity score analysis of 180 patients. *Lung Cancer.* 2018; 125:164-173.

30. Gauvin C, Krishnan V, Kaci I, et al. Survival Impact of Aggressive Treatment and PD-L1 Expression in Oligometastatic NSCLC. *Current oncology (Toronto).* 2021; 28(1):593-605.

31. Gorovets D, Ebner DK, Rava P, et al. Improving Patient Selection for Stereotactic Radiosurgery as the Initial Radiation Therapy Approach for Brain Metastases. *International journal of radiation oncology, biology, physics.* 2014; 90(1):S320-S320.

32. Gorovets D, Rava P, Ebner DK, et al. Predictors for long-term survival free from whole brain radiation therapy in patients treated with radiosurgery for limited brain metastases. *Frontiers in Oncology.* 2015; 5(MAY).

33. Gorovets DMD, Ayala-Peacock DMD, Tybor DJMPHP, et al. Multi-institutional Nomogram Predicting Survival Free From Salvage Whole Brain Radiation After Radiosurgery in Patients With Brain Metastases. *International journal of radiation oncology, biology, physics.* 2016; 97(2):246-253.

34. Gray PJ, Mak RH, Yeap BY, et al. Aggressive therapy for patients with non-small cell lung carcinoma and synchronous brain-only oligometastatic disease is associated with long-term survival. *Lung Cancer.* 2014; 85(2):239-244.

35. Griffioen GHMJ, Toguri D, Dahele M, et al. Radical treatment of synchronous oligometastatic non-small cell lung carcinoma (NSCLC): Patient outcomes and prognostic factors. *Lung Cancer.* 2013; 82(1):95-102.

36. Guo G, Lambert P, Ahmed N, et al. Local treatment improves survival in NSCLC patients with synchronous brain oligometastases. *International Journal of Radiation Oncology Biology Physics.* 2014; 90(5 SUPPL. 1):S52.

37. Harat M, Blok M, Kowalewska J, Miechowicz I. Stereotactic radiosurgery of brain metastasis in patients with a poor prognosis: Effective or overtreatment? *Cancer Management and Research.* 2020; 12:12569-12579.

38. Hirschmann E, Zoske H, Weissmann T, et al. Predictors of long-term survival following radical treatment of oligometastatic NSCLC with cerebral metastases. *Strahlentherapie und Onkologie.* 2018; 194(1 Supplement 1):43.

39. Inoue T, Katoh N, Aoyama H, et al. Clinical outcomes of stereotactic brain and/or body radiotherapy for patients with oligometastatic lesions. *Japanese Journal of Clinical Oncology.* 2010; 40(8):788-794.

40. Kaba E, Yardimci EH, Kakuturu J, Toker A. In Spite of Curative Radical Pulmonary Procedures, Lesser Pulmonary Resection Shows More Favorable Prognosis in Surgically Treated NSCLC With Synchronous Isolated Cranial Oligometastases. *Frontiers in surgery.* 2021; 8:645870.

41. Karlovits BJ, Quigley MR, Karlovits SM, et al. Stereotactic radiosurgery boost to the resection bed for oligometastatic brain disease: Challenging the tradition of adjuvant whole-brain radiotherapy. *Neurosurgical Focus.* 2009; 27(6):E7.1-E7.6.

42. Kocher M, Soffietti R, Abacioglu U, et al. Adjuvant Whole-Brain Radiotherapy Versus Observation After Radiosurgery or Surgical Resection of One to Three Cerebral Metastases: Results of the EORTC 22952-26001 Study. *Journal of Clinical Oncology.* 2010; 29(2):134-141.

© 2023 Li AY et al. *JAMA Network Open.*

43. Loi M, Mazzella A, Mansuet-Lupo A, et al. Synchronous Oligometastatic Lung Cancer Deserves a Dedicated Management. *Annals of Thoracic Surgery.* 2019; 107(4):1053-1059.

44. Lopez Guerra JL, Gomez D, Zhuang Y, et al. Prognostic impact of radiation therapy to the primary tumor in patients with non-small cell lung cancer and oligometastasis at diagnosis. *International journal of radiation oncology, biology, physics.* 2012; 84(1):e61-e67.

45. Macchia G, Ferro M, Deodato F, et al. OC-0058: SIB-IMRT in patients with < 5 brain metastases: phase I trial final results (ISIDE-BM-1). *Radiotherapy and oncology.* 2015; 115:S28-S29.

46. Mitchell KG, Farooqi A, Ludmir EB, et al. Improved Overall Survival With Comprehensive Local Consolidative Therapy in Synchronous Oligometastatic Non–Small-Cell Lung Cancer. *Clinical lung cancer.* 2020; 21(1):37-46.e37.

47. Mitin T, Chen Y-H, Catalano PJ, Floyd SR, Kasper EM, Mahadevan A. The role of systemic disease status in treatment outcomes for patients with newly diagnosed brain oligometastases and treated with stereotactic radiosurgery alone. *Journal of radiation oncology.* 2013; 3(1):43-48.

48. Naqash AR, Jonnalagadda S, Macherla S, et al. P1.04-60 Impact of Metastatic Location on Survival in Stage-IV Non-Small Cell Lung Cancer (NSCLC) Treated with Immunotherapy. *Journal of Thoracic Oncology.* 2019; 14(10 Supplement):S464.

49. Navarria P, Clerici E, Franzese C, et al. Can HSRS on tumor bed replace WBRT in resected brain metastases? Results of a phase II study. *Radiotherapy and Oncology.* 2019; 133(Supplement 1):S380.

50. Nieder C, Mannsaker B, Yobuta R. Late Brain Oligometastases Diagnosed at Least 36 Months after Cancer Detection are Associated with Favorable Survival Outcome. *Cureus.* 2020; 12(1):e6553.

51. Nieder C, Hintz M, Popp I, Bilger A, Grosu AL. Long-term survival results after treatment for oligometastatic brain disease. *Reports of Practical Oncology and Radiotherapy.* 2020; 25(3):307-311.

52. Niibe Y, Nishimura T, Inoue T, et al. Oligo-recurrence predicts favorable prognosis of brain-only oligometastases in patients with non-small cell lung cancer treated with stereotactic radiosurgery or stereotactic radiotherapy: A multi-institutional study of 61 subjects. *BMC Cancer.* 2016; 16(1).

53. Nikitas J, Roach M, Robinson C, et al. Treatment of oligometastatic lung cancer with brain metastases using stereotactic radiosurgery (SRS) and stereotactic body radiation therapy (SBRT). *Clinical and translational radiation oncology.* 2020; 21:32-35.

54. Pikin O, Kolbanov K, Glushko V, Amiraliev A, Vursol D, Kartoveshenko A. Oligometastases in NSCLC patients: Is surgery justified? *Journal of Thoracic Oncology.* 2011; 6(6 SUPPL. 2):S612-S613.

55. Pikin O, Glushko V, Kolbanov K, et al. Does surgery have real benefit in resectable oligometastatic NSCLC? *Journal of Thoracic Oncology.* 2017; 12(1 Supplement 1):S779.

56. Raez LE, Botero A, Castillo M, Castellon I, Hunis B, Falchook A. Effect of oligometastatic disease management with stereotactic body radiation therapy on survival in patients with metastatic lung cancer. *Journal of Clinical Oncology.* 2019; 37(15_suppl):e13582-e13582.

© 2023 Li AY et al. *JAMA Network Open.*

57. Rogers LR, Rock JP, Sills AK, et al. Results of a phase II trial of the GliaSite Radiation Therapy System for the treatment of newly diagnosed, resected single brain metastases. *Journal of neurosurgery.* 2006; 105(3):375-384.

58. Salvador Coloma C, Lorente D, Mendez J, Juan-Vidal O, Escoin C. Long-Term Survival for Brain-Only Oligometastatic NSCLC Patients Treated with Ablative Therapy (AT): Prognostic Factors. *Journal of Thoracic Oncology.* 2018; 13(10 Supplement):S941.

59. Sato J, Horinouchi H, Goto Y, et al. Long-term survival without surgery in NSCLC patients with synchronous brain oligometastasis: systemic chemotherapy revisited. *Journal of thoracic disease.* 2018; 10(3):1696-1702.

60. Shibata K, Iwasa K, Takanaka T, et al. Curative thoraco-systemic therapy plus local treatment to the brain for extensive disease-small-cell lung cancer with metastasis only to the brain. *Japanese Journal of Clinical Oncology.* 2019; 49(7):687-690.

61. Shirasawa M, Fukui T, Kusuhara S, et al. Prognostic differences between oligometastatic and polymetastatic extensive disease-small cell lung cancer. *PLoS ONE.* 2019; 14(4):e0214599.

62. Song Y-Q, Wang N, Li X, et al. Treatment patterns and survival after 18F-fluorodeoxyglucose positron emission tomography/computed tomography-guided local consolidation therapy for oligometastatic non-small cell lung cancer: a two-center propensity score-matched analysis. *Journal of Cancer Research and Clinical Oncology.* 2020; 146(4):1021-1031.

63. Suzuki K, Shiono S, Hasumi T, et al. Clinical significance of bifocal treatment for synchronous brain metastasis in T1-2 non-small-cell lung cancers: JNETS0301. *General Thoracic and Cardiovascular Surgery.* 2021; 69(6):967-975.

64. Wang Z, Gao SG, Xue Q, et al. Surgery of primary non-small cell lung cancer with oligometastasis: Analysis of 172 cases. *Journal of Thoracic Disease.* 2018; 10(12):6540-6546.

65. Xu Y, Lv X, Liu Y, Dong B. Brain oligometastases in NSCLC: Therapeutic effect and prognostic factors of WBRT combined with intracarotid infusion of elemene. *Journal of Thoracic Oncology.* 2018; 13(4 Supplement 1):S125-S126.

66. Yamaguchi M, Edagawa M, Suzuki Y, et al. Pulmonary Resection for Synchronous M1b-cStage IV Non-Small Cell Lung Cancer Patients. *Annals of Thoracic Surgery.* 2017; 103(5):1594-1599.

67. Yegya-Raman N, Aisner J, Kim S, et al. Synchronous Oligometastatic Non-small Cell Lung Cancer Managed With Curative-Intent Chemoradiation Therapy: Long-term Outcomes From a Single Institution. *Advances in Radiation Oncology.* 2019; 4(3):541-550.

68. Zhang C, Wang L, Li W, et al. Surgical outcomes of stage IV non-small cell lung cancer: A single-center experience. *Journal of Thoracic Disease.* 2019; 11(12):5463-5473.

69. Guyot P, Ades AE, Ouwens MJNM, Welton NJ. Enhanced secondary analysis of survival data: reconstructing the data from published Kaplan-Meier survival curves. *BMC medical research methodology.* 2012; 12(1):9-9.

© 2023 Li AY et al. *JAMA Network Open.*
