# Supplementary material for: Association of Brain Metastases With Survival in Patients With Limited or Stable Extracranial Disease: A Systematic Review and Meta-analysis
Source: JAMA Netw Open. 2023 Feb 23;6(2):e230475. doi: 10.1001/jamanetworkopen.2023.0475 (PMC9951042; doi:10.1001/jamanetworkopen.2023.0475)
Supplement: Supplement 2. — Data Sharing Statement [file jamanetwopen-e230475-s002.pdf]

Data Sharing Statement

Li. Association of Brain Metastases With Survival in Patients With Limited or Stable Extracranial Disease. *JAMA Netw Open*. Published February 23, 2023.
doi:10.1001/jamanetworkopen.2023.0475

Data
Data available: Yes
Data types: Deidentified participant data
How to access data: https://www.dropbox.com/sh/ili4y7jlbxdus6q/AADfadR6rKH6k728-9yg688Za?dl=0
When available: With publication

Supporting Documents
Document types: Statistical/analytic code
How to access documents: https://www.dropbox.com/sh/ili4y7jlbxdus6q/AADfadR6rKH6k728-9yg688Za?dl=0
When available: With publication

Additional Information
Who can access the data: Any academic affiliate who wishes to use the dataset for meta-analyses or subgroup analyses
Types of analyses: Meta-analyses, subgroup analyses
Mechanisms of data availability: None
